# 3-Hydroxy coumarin demonstrates anti-biofilm and anti-hyphal efficacy against *Candida albicans* via inhibition of cell-adhesion, morphogenesis, and virulent genes regulation

**DOI:** 10.1038/s41598-023-37851-1

**Published:** 2023-07-19

**Authors:** T. J. Sushmitha, Meora Rajeev, Vellaisamy Kathirkaman, Singh Shivam, Toleti Subba Rao, Shunmugiah Karutha Pandian

**Affiliations:** 1 Department of Biotechnology, Alagappa University, Science Campus, Karaikudi, Tamil Nadu 630 003 India; 2 School of Arts and Sciences, Sai University, OMR, Paiyanur, Tamil Nadu 603105 India; 3 Present Address: Department of Biological Sciences and Bioengineering, Inha University, Inharo 100, Incheon, 22212 Republic of Korea

**Keywords:** Antimicrobials, Bacteria, Biofilms, Environmental microbiology

## Abstract

*Candida albicans*, a common fungus of human flora, can become an opportunistic pathogen and causes invasive candidiasis in immunocompromised individuals. Biofilm formation is the prime cause of antibiotic resistance during *C. albicans* infections and treating biofilm-forming cells is challenging due to their intractable and persistent nature. The study intends to explore the therapeutic potential of naturally produced compounds by competitive marine bacteria residing in marine biofilms against *C. albicans* biofilm. To this end, 3-hydroxy coumarin (3HC), a compound identified from the cell-free culture supernatant of the marine bacterium *Brevundimonas abyssalis*, was found to exhibit anti-biofilm and anti-hyphal activity against both reference and clinical isolates of *C. albicans*. The compound demonstrated significant inhibitory effects on biofilms and impaired the yeast-to-hyphal transition, wrinkle, and filament morphology at the minimal biofilm inhibitory concentration (MBIC) of 250 µg mL^−1^. Intriguingly, quantitative PCR analysis of 3HC-treated *C. albicans* biofilm revealed significant downregulation of virulence genes (*hst7, ume6, efg1, cph1, ras1, als1*) associated with adhesion and morphogenesis. Moreover, 3HC displayed non-fungicidal and non-toxic characteristics against human erythrocytes and buccal cells. In conclusion, this study showed that marine biofilms are a hidden source of diverse therapeutic drugs, and 3HC could be a potent drug to treat *C. albicans* infections.

## Introduction

*Candida albicans* is a commensal polymorphic fungus that generally inhabits human skin and the surface of mucous membranes. However, under certain conditions such as immune system failure and microbiota imbalance, this fungal species can become pathogenic and cause superficial oral and vaginal candidiasis, as well as invasive candidiasis^1,2^. Studies have recorded nearly 50,000 deaths annually due to invasive candidiasis, with *C. albicans* being the most reported species, accounting for 40–50% of the cases^3,4^. The prime factor that is conducive to the pathogenesis of *C. albicans* is its ability to form biofilm on both biotic and abiotic surfaces, followed by other major virulence traits, such as yeast-to-hyphal transitions, filamentous morphology, wrinkle morphology, and secretion of proteolytic and lipolytic enzymes^5^. Further, virulence traits, including adhesion, hyphal, and biofilm formation enable *C. albicans* to access deep tissues for systemic infections. The combat against the virulence traits of *C. albicans* involves four major classes of antifungal drugs, including polyenes, echinocandins, nucleoside analogues, and azoles^6,7^. However, biofilm formation by *C. albicans* has been found to gain genetic resistance against most of the currently used antifungal drugs. Therefore, there is a critical need for alternative therapeutic agents to fight against biofilm-mediated infections and to conquer the limitations of current antifungal therapies. In this milieu, the search for novel anti-*Candida* compounds is imperative, particularly from natural environments such as marine environment.

The exploitation of natural products, such as compounds derived from marine bacteria, offers new prospects for the development of novel drug entities. In natural environmental conditions, marine biofilms consist of multispecies bacterial communities in close proximity, with large metabolite and protein exchange, as well as coordinated lifestyles. These complex interactions may benefit or harm the interacting bacterial groups and aid the bacterial consortia in maintaining ecological equilibrium. For example, resident bacterial communities establish either cooperative (beneficial) or competitive (harmful) interactions, which affect biofilm succession, biomass, and resistance to stress. Several independent studies have shown that surface-associated bacteria produce bioactive compounds with clinical importance, including antibiotics and anti-biofilm agents^8–10^. These bioactive agents enable to override the competition for surface colonization. We hypothesized that some of these bioactive compounds can be advantageously used to inhibit human pathogens.

A plethora of studies have identified compounds derived from the marine environment as potential inhibitors of biofilm formation in bacterial^11–13^ and fungal pathogens^14–21^. However, these compounds are typically associated with seawater or sediment, where the residing bacterial community experiences less competition. Compounds originating from naturally competitive environments, such as marine biofilms, are more likely to possess greater potential to inhibit the virulence of other organisms.

In this investigation, we obtained potent biofilm-forming bacterial isolates from the early-stage biofilm formed on three different artificial surfaces (stainless steel, high-density polyethylene, and titanium) immersed in the intake area of a coastal located power plant^22^. The cell-free culture supernatant (CFCS) of the bacterial isolates was screened for their potent anti-biofilm efficacy against *C. albicans* and the active compounds were identified using HR-LCMS analysis from the selected CFCS. We then examined the anti-biofilm activity of the lead bioactive compound against *C. albicans* and explored the underlying mechanisms. The major objective of this research work is to disrupt the multicellularity of *C. albicans* biofilm so that the effectiveness of the host defense mechanism could be re-established.

## Materials and methods

### Ethics statement

In the present study, we collected human buccal epithelial cells (HBECs) and human erythrocytes by gently swabbing both sides of the mouth using sterile cotton swabs and by venipuncture, respectively. Both samples were collected from healthy individuals and written consent was obtained after explaining the purpose of sample collection and research in their native language. The experimental protocol for procuring HBECs and blood was approved by the Institutional Ethics Committee, Alagappa University, Karaikudi (IEC Ref. No.: IEC/AU/2018/5). All methods were carried out in accordance with relevant guidelines and regulations and informed consent was obtained from all subjects and/or their legal guardian(s).

### *C. albicans* strains and their growth conditions

All experiments in the present study were performed using a reference *C. albicans* strain (*C. albicans* 90028) from the ATCC, and three clinical isolates (designated as CI 1, CI 2 and CI 3), which were originally collected from the *Candida*-infected patients at a government hospital in Coimbatore, Tamil Nadu, India. The three clinical isolates were identified at species level in our previous study^23^ based on internal transcribed spacer (ITS) gene sequencing (GenBank accession numbers: MF423465–MF423467). The reference *C. albicans* strain 90028 and other clinical isolates were maintained in yeast extract peptone dextrose (YEPD; 1% yeast extract, 2% peptone, and 2% dextrose) agar plates at 4 ℃. A 30% glycerol stock was also maintained and stored in an ultra-deep freezer at − 80 ℃ temperature for further use. For routine culturing and in vitro planktonic cell assays, YEPD broth was used, and the culture was grown at 37 ℃ with constant shaking at 160 rpm. A specialized Spider medium encompassing 1% mannitol, 0.2% dipotassium hydrogen phosphate, and 1% nutrient broth was used to perform biofilm experiments. Further, RPMI medium (HiMedia Ltd., India) and Spider medium supplemented with 10% Fetal Bovine Serum (FBS) were used for all yeast-to-hyphal and hyphal-to-yeast transition experiments^13,24^. In order to simulate the oral environment and investigate the potential anti-biofilm properties, the study utilized artificial saliva and the composition was prepared following the methodology outlined in the previous literatures^25,26^.

### Collection and growth conditions of marine biofilm-forming bacterial isolates

The bacterial isolates utilized in this study were originally isolated from the early-stage marine biofilms developed on various artificial surfaces, such as stainless steel, high-density polyethylene, and titanium, in the intake area of a coastal located nuclear power plant, located on the southern coast of India^22^. Briefly, the biofilms developed on the artificial surfaces were scraped using sterile toothbrushes and suspended in sterile seawater. The suspended biofilms were transported to the laboratory under ice-cold conditions and immediately spread-plated on various growth media, including Zobell marine agar 2216 (ZMA, HiMedia Ltd., India), Luria Bertani Agar, and artificial sea salt agar (HiMedia Ltd., India), to obtain a wide range of culturable bacterial isolates. All the plates were incubated at 30 ℃ for seven days and the growth of bacterial isolates was checked every 24 h. Each newly grown bacterial morphotype was picked using sterile toothpicks and subcultured on ZMA. Axenic cultures of the selected morphotypes were further obtained using quadrant streaking and preserved as glycerol stocks at − 80 ℃.

### Screening of marine bacteria with anti-biofilm activity against *C. albicans*

A collection of 60 bacterial isolates obtained from the natural marine biofilm was screened for their ability to inhibit biofilm formation by *C. albicans*, as described earlier^23^. Briefly, bacterial isolates were grown in Zobell marine broth (ZMB, HiMedia Ltd., India) overnight, and the cell-free culture supernatants (CFCSs) were collected by centrifugation. The collected CFCSs were filtered individually using 0.22-µm syringe filters. Entire screening was performed in experimental triplicates using 96-well microtiter plates (MTP). Filtered CFCSs were added (10% v/v) to the Spider medium containing an adequate density *of C. albicans* strain 90028 cells (10^6^ CFU/ml). The cultured plates were then incubated at 37 ºC for 48 h to facilitate biofilm formation. Alongside this experimental group, suitable control measures, including an appropriate control, vehicle control, and negative control, were also implemented.

After the incubation period, the planktonic cell fraction was discarded, and MTP were gently washed with sterile saline to remove any loosely adhered cells. The plates were then air-dried, and 0.4% crystal violet (CV) solution was added to stain the biofilm formed on the inner surface of the wells. The CV bound to biofilm-forming cells was resuspended in 200 µL of 30% acetic acid solution, the optical density (OD) was measured at 570 nm, and expressed as biofilm biomass^27^. Here, wells containing *C. albicans* cells but without any bacterial CFCSs treatment were considered as control to determine the biofilm-biomass reduction. Based on the screening results, one marine bacterial isolate, 6HZ5 (the fifth identified isolate from a 6-day-old biofilm formed on high-density polyethylene coupon and grown on Zobell marine agar), showed promising anti-biofilm activity and was therefore selected for further investigation.

### Identification and phylogenetic affiliation of lead bacterial isolate

Genomic DNA was extracted from the selected bacterial isolate using the DNeasy PowerLyzer Microbial Kit (Qiagen, Cat. No.: 12255-50) and polymerase chain reaction (PCR) was performed to amplify the 16S rRNA gene using universal bacterial primers: 27F (5′-AGAGTTTGATCCTGGCTCA-3′) and 1390R (5′-GACGGGCGGTGTGTACAA-3′) as described in our earlier studies^27^. The amplicons were sequenced using BigDye Terminator v3.1 Cycle Sequencing Kit (Thermo Fisher Scientific, India) on an Applied Biosystems 3130 Genetic Analyzer (Applied Biosystems, USA). Trimmed and edited sequences obtained from Sanger sequencing were assembled in CAP3 Contig Assembly Program and the concatenated sequence was compared with available 16S rRNA gene sequences in the GenBank database (https://blast.ncbi.nlm.nih.gov/Blast.cgi) using the BLAST algorithm^27,28^. Finally, a nearly full-length 16S rRNA gene sequence was submitted to NCBI-GenBank under accession code MZ357100. Further, a phylogenetic tree based on obtained 16S rRNA gene sequence in this study and other reference sequences was constructed as described earlier^29^ using maximum-likelihood method (1000 bootstrap replicates) in the MEGA (version 11) software^30^.

### High-resolution liquid chromatography–mass spectrometry (HRLC–MS) analysis of crude bacterial extract

The selected bacterial isolate (6HZ5), which showed potent anti-biofilm activity against *C. albicans* was grown in Zobell marine broth (ZMB, HiMedia Ltd., India) at 30 ºC under shaking conditions (120 rpm) for 48 h. After incubation, the bacterial suspension was centrifuged to collect CFCS, and the compounds were extracted using twice the volume of ethyl acetate. The resulting extract was dried at 55 °C and dissolved in sterile distilled water. Here, the crude extract was confirmed for its anti-biofilm activity against *C. albicans* and maintained at 30 °C for further analysis. Subsequently, the partially purified extract was subjected to LC–MS analysis using Agilent Orbitrap mass analyzer HRLC-MS System with a dual Agilent Jet Stream Electron Ionization source (AJS ESI) ion source mode^31^. After each sample, a blank was run. A mobile phase consisting of 0.1% v/v formic acid in water (A) and 10% water with 90% acetonitrile and 0.1% formic acid (B) was used. The gradient elution program was as follows: 5% B isocratic for 5 min, from 5 to 95% B for 20 min, and 95% B isocratic for 5 min. The flow rate and maximum pressure limit were set at 300 µL min^−1^ and 12,000 psig, respectively. Sample (3 µL volume) was injected after needle wash. Quadrupole-Time of Flight (Q-TOF) conditions were as follows: gas flow −11 mL min^−1^ at 300 °C, nebulizer pressure—35 psig, nozzle voltage—1 kV, and fragmentor voltage—175 V. Acquisition mode was set between 60 and 1000 *m/z* range at a scan rate of 1spectra s^−1^_._

### Screening of identified compounds for their anti-biofilm potential against reference *C. albicans* 90028

LCMS analysis confirmed the presence of a total of 80 compounds in the selected bacterial CFCS. The abundant compounds that were available commercially were procured and tested for their ability to *C. albicans* inhibit biofilm. The seven compounds tested in our study were as follows: 11-amino undecanoic acid, Salfredin 6,8-Docosanedione, 7-acetoxy-4-methyl coumarin, sulfadimidine, Moracin D and 3-hydroxy coumarin (3HC). All the compounds were procured from Tokyo Chemical Industry (TCI), Japan and all had a purity of > 98.0%. To test their anti-biofilm efficacy, stock solutions of procured compounds were prepared with respect to their solubility in different solvents (methanol, ethanol, and water). These compounds were tested against *C. albicans* in a 24-well MTP using YEPD and Spider media for planktonic and biofilm cells, respectively, as described earlier^32^. Each well was loaded with 1 mL of appropriate growth medium, 10^6^ CFU/mL of *C. albicans* 90028 cells*,* and selected compounds at 500 µg mL^−1^ concentration. The MTPs were incubated at 37 ℃ for 24 h and 48 h for planktonic and biofilm-forming cells, respectively. To assess the planktonic cell density, the 24 h incubated plates were read at 600 nm. On the other hand, biofilm-forming cells underwent a similar experimental procedure to that of bacterial screening mentioned above. Among the tested compounds, 3HC exhibited potential anti-biofilm activity against all the *C. albicans* strains without exerting any influence on planktonic cells, and therefore selected for further study.

## Impact of 3HC on planktonic and biofilm-forming *C. albicans* cells

### Minimal inhibitory concentration (MIC) ascertainment of 3HC

A stock solution (20 mg mL^−1^) of the compound was prepared in ethanol and stored at 30 ℃ for further use. To account the effect of solvent, an appropriate vehicle control was maintained in all experiments. The anti-biofilm activity of 3HC against *C. albicans* strains was assessed using the standard microbroth dilution method suggested by the Clinical and Laboratory Standards Institute (CLSI, 2022; document M100), and as detailed in earlier studies previously described^23^. To briefly describe, 1% inoculum of overnight grown *C. albicans* strains comprising 10^6^ CFU/ml cells was inoculated in 1 mL of YEPD broth containing varying 3HC concentrations (ranging from 62.5 to 2000 µg mL^−1^) and incubated for 24 h at 37 ℃. Here, wells without 3HC treatment served as control, and wells containing only YEPD medium served as a negative control. After incubation, cell density was quantified by measuring the absorbance at 600 nm using a spectrophotometer (Spectra Max 3, Molecular Devices, USA). In addition, 5 µL of cells from each well was also spotted on YEPD agar Petri plates to observe growth effects. The plates were imaged after 24 h of incubation at 37 ℃.

### Influence of 3HC on metabolic viability of *C. albicans*

Metabolic viability of control and treated *C. albicans* cells was measured using Alamar blue [Resazurin (7-Hydroxy-3H-phenoxazin-3-one 10-oxide)] method^33^. Briefly, after treating the cells with 3HC for 24 h, the treated and control cells were centrifuged at 8000*g* for 10 min and gently washed thrice with phosphate buffer saline (PBS, pH 7.4). The washed cells were resuspended in 1 mL PBS and then 10 µL of Alamar blue (10 mg/mL stock) was added and incubated at 37 ℃ in dark for 18–24 h for the fluorescence development. Alamar blue in PBS without the cells served as a negative control. After the incubation period, the cells were centrifuged, and the fluorescence intensity of the supernatant was measured at 560 nm (excitation) and 590 nm (emission).

### Determination of minimal biofilm inhibitory concentration (MBIC) of 3HC

The standard CV (0.4%) quantification method (as followed for anti-biofilm screening) was used to determine the MBIC of *C. albicans* strains^32^. In short, 10^6^ cells of *C. albicans* were inoculated in 1 mL Spider medium and artificial saliva containing varying 3HC concentrations (62.5 to 2000 µg mL^−1^) in 24-wells MTP. The MTP was incubated at 37 °C for 48 h. After incubation, the planktonic cells were discarded, and the wells were washed carefully to remove loosely adhered cells. The plates were then stained using CV (0.4%) for 10 min. The excess stain was washed out and the biofilm-bound stain was dissolved in 1 mL of 30% acetic acid. The biofilm inhibition was measured at 570 nm, and a minimum of 80% inhibition was considered as MBIC. The percentage of biofilm inhibition was determined by means of the following formula:

$${\text{Biofilm inhibition }}\left( \% \right)\, = \,\left[ {\left( {{\text{Control}}_{{{\text{OD}}}} \, - \,{\text{Treated}}_{{{\text{OD}}}} } \right)/{\text{Control}}_{{{\text{OD}}}} } \right]\, \times \,{1}00.$$

### Effects of 3HC on the metabolic activity of *C. albicans* biofilms

The fluorescein diacetate (FDA) staining dye was used to evaluate and quantify the viable biofilm biomass of *C. albicans* strains^34,35^. Briefly, 10^6^ cells of *C. albicans* strains were allowed to form biofilm in 24-well MTP in the presence and absence of 3HC for 48 h. After incubation, the planktonic cells were discarded, and 0.2 mg mL^−1^ of FDA and RPMI medium were mixed in 1:1 (v/v) freshly and transferred to the wells of 24 MTP. The plates were incubated at 37 ℃ for 1 h in dark. The fluorescence intensity was measured at an excitation of 488 nm and emission of 530 nm. Further, the plate was rinsed gently with PBS to remove excess stains and observed under a fluorescence microscope.

### Field emission scanning electron microscopy (FESEM) analysis

To further evaluate the effectiveness of 3HC on biofilm and hyphal reduction, the control and 3HC-treated *C. albicans* cells (at various concentrations) were subjected to FESEM analysis, in accordance with our earlier study^22^. Briefly, the 10^6^ cells of *C. albicans* 90028 were allowed to form biofilms on a glass slide for 48 h in the presence and absence of 3HC. After incubation, the slides were gently washed with PBS to remove loosely adhered cells and fixed with freshly prepared 2% glutaraldehyde at 4 ℃ for 3 h. The samples were then dehydrated by sequential exposure to increasing concentrations of ethanol (20%, 40%, 60%, 80%, and 100%) for 10 min each. Finally, the cells were air-dried and visualized under FESEM (SUPRA 55VP; Carl Zeiss, Germany).

## Impact of 3HC on virulence traits of *C. albicans*

### Filamentous morphology

The effect of various 3HC concentrations on filament growth of *C. albicans* was evaluated on YEPD agar supplemented with 10% FBS^23,24,32^. Concisely, the required concentrations of 3HC was added to autoclaved YEPD agar and allowed to solidify. A culture suspension (5 μL containing ~ 10^6^ cells of *C. albicans*) was spotted onto the center of solidified agar. YEPD agar lacking 3HC served as control. The spots were allowed to dry, and the plates were incubated at 37 °C for 5 days for the filament morphology to appear. The hyphal-like protrusions (filament) observed were then documented using a high-resolution charge-coupled device (CCD) camera (GelDoc XR+, Bio-Rad).

### Wrinkle morphology

The colony morphology of *C. albicans* was evaluated in a similarly manner to the filamentous morphology. YEPD agar with various concentrations of 3HC was considered as the treatment group, while YEPD agar without 3HC as the control. A culture of *C. albicans* (5 µL) was spotted in the center of the agar plate and incubated at 37 °C for 72 h. The changes in morphology were documented using a gel documentation system.

### Yeast-to-hyphal transition

To investigate the effect of 3HC on yeast-to-hyphal phase transition, 1% of 10^6^ cells of *C. albicans* was inoculated into YEPD medium supplemented with 10% FBS^36–38^. Treatment groups received 3HC at different concentrations, while the control group had the same composition but without 3HC. The inoculated tubes were incubated at 37 °C with constant shaking at 160 rpm. After 4 h of incubation, the phase transition was documented using a light microscope.

### Hyphal-to-yeast transition

Similarly, the reversal of hyphal-to-yeast cells was also studied by inducing the formation of hyphae from yeast cells of *C. albicans* through incubation in RPMI medium for 4 h at 37 °C^38^. After incubation, 3HC at different concentrations was used for treatment, and the reverse transition was observed under a light microscope after 2 h, 4 h and 6 h of incubation.

### Efficacy of 3HC on mature biofilm disintegration

To test the efficacy of 3HC to disintegrate the mature biofilm, *C. albicans* was allowed to form biofilm in Spider medium for 48 h. After incubation, the spent medium was refreshed with fresh Spider medium supplemented with varying 3HC concentrations. Wells without 3HC treatment were considered as controls. The MTP was further incubated for 3 h to facilitate 3HC action on mature *C. albicans* biofilm. The biofilm removal was recorded using the standard 0.4% CV staining method and light microscopy.

### Effect of 3HC on virulence gene expression of *C. albicans*

The effect of 3HC on the gene expression profile of reference *C. albicans* strain 90028 was analyzed using quantitative real-time PCR (qRT-PCR), following the method described earlier^23,32^. Concisely, RNA was isolated from both control and 3HC-treated cells (at MBIC) using TRIzol method (TRI reagent, Sigma-Aldrich, India). The isolated RNA was quality-checked and quantified using agarose gel electrophoresis and NanoDrop spectrophotometer (NanoDrop Technologies, USA), respectively, and then immediately converted to cDNA using a high-capacity cDNA Reverse Transcription kit (Applied Biosystems, USA). After this step, qRT-PCR was performed using SYBR Green master mix (Applied Biosystems, United States). Primers of different genes were added individually with the SYBR Green master mix in a predefined ratio to make a total reaction volume upto 10 µL. The genes used in this study are considered crucial for *C. albicans* pathogenicity and are listed in Table 1.

**Table 1:** List of candidate genes used in this study for quantitative real-time PCR, their primer details, and function in the virulence and pathogenicity of *C. albicans*.

Gene	Function	Primer sequence (5ʹ–3ʹ)	References
*als3*	Agglutinin-like protein that is vital for adhesion to oral mucosa and epithelial cells. Mediates yeast aggregation that acts as a primary step in biofilm formation	F: CAACTTGGGTTATTGAAACAAAAACA R: AGAAACAGAAACCCAAGAACAACC	Green et al., 2006^69^
*hwp1*	Hyphal wall protein gene essential for hyphal elongation	F: GCTCCTGCTCCTGAAATGAC R: CTGGAGCAATTGGTGAGGTT	Carlisle and Kadosh, 2013^70^
*ume6*	Transcriptional activator of filamentous growth. Regulates hyphal elongation and germ tube formation	F: ACCACCACTACCACCACCAC R: TATCCCCATTTCCAAGTCCA	
*eap1*	Associated with cell adhesion and filamentation. Initiates adhesion to polystyrene and epithelial cells	F: TGTGATGGCGGTTCTTGTTC R: GGTAGTGACGGTGATGATAGTGACA	Fox et al., 2013^71^
*efg1*	A transcription factor that regulates switch between white and opaque cells and cell wall dynamics. Essential for biofilm formation and filamentous growth	F: GCCTCGAGCACTTCCACTGT R: TTTTTTCATCTTCCCACATGGTAGT	Kumamoto and Vinces, 2005^72^
*cph1*	Transcription factor involved in pseudohyphal and hyphal formation and phenotypic switching	F: TATGACGCTTCTGGGTTTCC R: ATCCCATGGCAATTTGTTGT	
*ras1*	Mediates cell adhesion, induces filamentous growth, regulates phenotypic switching	F: CCCAACTATTGAGGATTCTTATCGTAAA R: TCTCATGGCCAGATATTCTTCTTG	Inglis and Sherlock, 2013^73^
*hst7*	Filamentation required for opaque mating or white biofilm formation	F: TCATCAGCTTCTTCTATAC R: TATTGAGGAAATGACAGTT	Köhler and Fink, 1996^74^

The expression profile of the genes involved in biofilm formation and virulence of *C. albicans* was studied with the Applied Biosystems^®^ 7500 Real-Time PCR system. The housekeeping ITS gene with a size of ~540 bp was used as an internal control and the relative gene expression of control and treated cells was determined using ΔΔCT method^39^.

## Cytotoxicity analysis of 3HC against human buccal epithelial cells (HBECs) and human erythrocytes cells (HECs)

### Effect of 3HC on HBECs

Healthy volunteers were recruited, and HBECs were collected by gently rubbing both sides of the inner cheeks with a sterile cotton swab. The swab was suspended in sterile PBS and vortexed. All the collected cells were pooled and centrifuged at 3000*g* for 10 min. The pellet was washed thrice with PBS and resuspended in PBS. Cells were adjusted to ~ 10^5^ cells/mL using an automated cell counter (Countess II FL, Invitrogen, USA) and incubated with varying 3HC concentrations. Hydrogen peroxide was used as positive control and the cells without any treatment served as control. After 20 min of incubation at 37 ℃, the cells were stained with CV and the toxic effect was observed under a light microscope^32^.

### Effect of 3HC on HECs

Similar to HBECs, HECs were also collected from healthy volunteers. Here, 2 mL of blood was drawn through venipuncture and was added to a tube containing an anticoagulant (a pinch of EDTA). HECs were harvested by centrifugation (2000*g* at 10 min) and washed thrice with PBS. The cells were then equally diluted and treated with various concentrations of 3HC. In this experiment, 1% of Triton X-100 was used as a positive control to rupture HECs. The treated tubes were incubated for 1 h at 37 ℃. The tubes were centrifuged again after incubation and the supernatant was transferred to a 96-well MTP^40^.

### Statistical analyses

All the experiments were performed in at least three biological replicates, each with a minimum of three experimental replicates. The data shown are presented as the mean ± standard deviation (SD). Analysis of variance (ANOVA) was performed, followed by Duncan’s post-hoc test, using R to determine the significance within and among groups.

## Results

### Anti-biofilm potential of 6HZ5 culture supernatant against *C. albicans*

To obtain a potent biofilm inhibitor, CFCS of 60 marine bacterial isolates obtained from early-stage marine biofilms were screened for their ability to inhibit biofilm formation by *C. albicans* (Supplementary Fig. S1a–d). Of the screened bacterial isolates, 20 isolates showed moderate anti-biofilm activity, with one isolate (designated as strain 6HZ5) unveiled the strongest biofilm inhibition ability (> 95%) against *C. albicans* (Supplementary Fig. S1b). Furthermore, the results revealed that the CFCS of bacterial strain 6HZ5 did not affect the growth of *C. albicans* cells.

Interestingly, screening results showed that few bacterial isolates also had the potential to enhance biofilm formation. For example, bacterial isolates designated as 9SZ1, 9SZ2 and 9SA3 were found to enhance the biofilm-forming potential of *C. albicans* by 40% compared to their corresponding controls. After careful evaluation of overall screening results, we concluded that the CFCS of a marine bacterial strain 6HZ5 had the strongest anti-biofilm activity against *C. albicans* and therefore this isolate was selected further in our study. Based on the 16S rRNA gene sequence analysis, the selected bacterial isolate with potent anti-biofilm activity was identified as *Brevundimonas abyssalis* strain 6HZ5. Further, a phylogenetic tree was constructed using 32 reference 16S rRNA gene sequences of *Brevundimonas* spp. (Supplementary Fig. S2). The selected bacterium *B. abyssalis* strain 6HZ5 showed a close phylogenetic similarity with *B. canariensis*. These two bacteria also formed a separate clade from the other species of genus *Brevundimonas*.

### Identification of active compounds in CFCS of *B. abyssalis* strain 6HZ5

HR-LCMS analysis was used to identify the active compounds present in the CFCS. The identified compounds are listed in Supplementary Table S1. To proceed further, the predominant compounds such as 11-amino undecanoic acid, 6,8-docosanedione, 7-acetoxy-4-methyl coumarin, 3-hydroxy coumarin, Sulfadimidine, Moracin D and Salfredin were procured commercially and were screened for their anti-biofilm potential against *C. albicans*.

### Screening of identified potent anti-biofilm compounds against *C. albicans*

**Figure 1:**
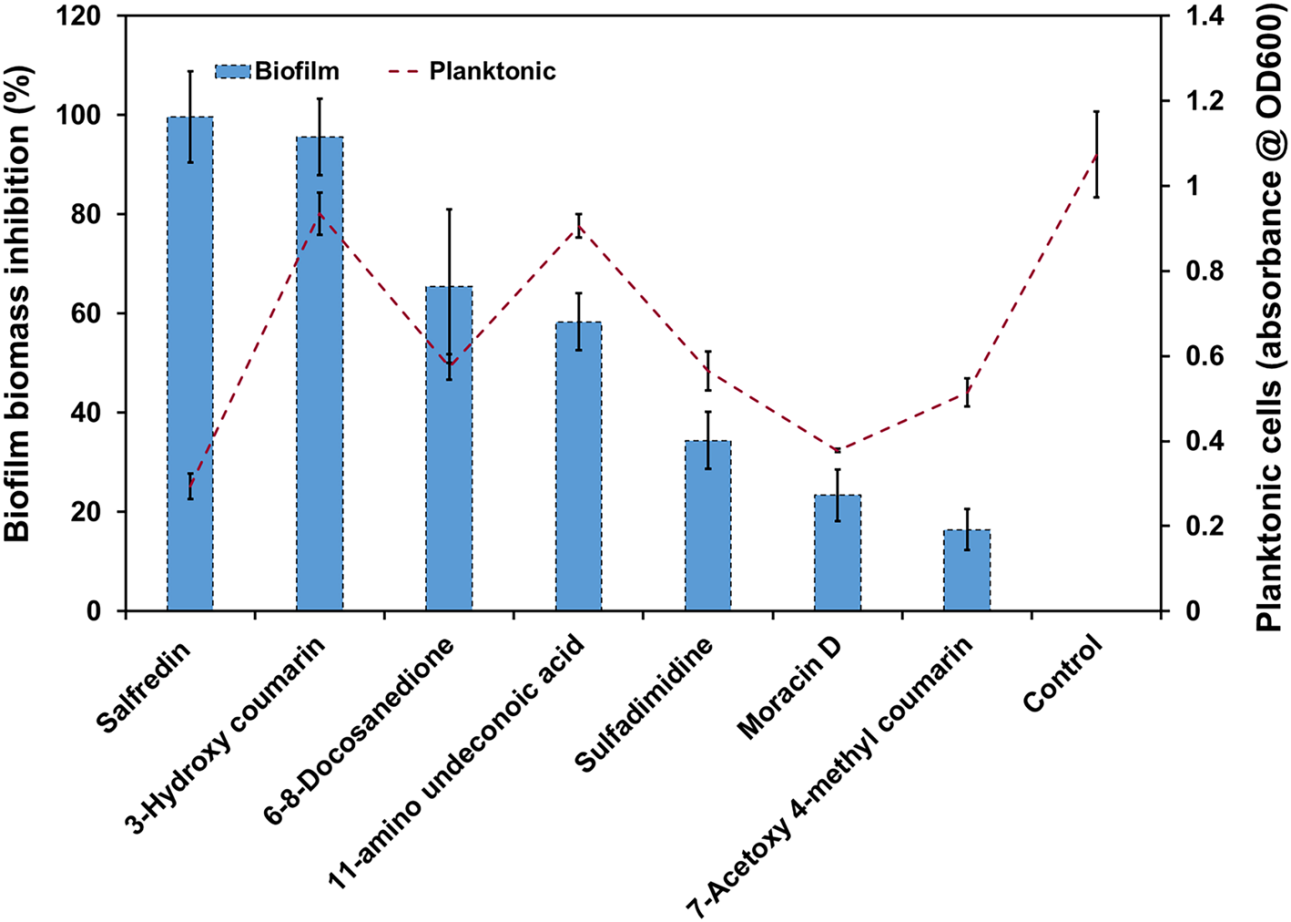
Screening of commercially available compounds identified through HR-LCMS analysis in cell-free culture supernatant (CFCS) of a marine bacterial isolate *Brevundimonas abyssalis* for their anti-biofilm potential against *C. albicans*. Bar graph depicts the biofilm inhibition (%) and the line graph shows planktonic cells absorbance at 600 nm. Two compounds namely 3-hydroxy coumarin (3HC) and 11-amino undeconoic acid displayed potent anti-biofilm activity against *C. albicans*. Error bars represent standard deviations from the mean of experimental triplicates.

The effect of the selected compounds was analyzed for their growth and biofilm inhibitory potential against *C. albicans* (Fig. 1). Of the screened seven compounds, six compounds namely 6–8-Docosanedione, 7-Acetoxy 4-methyl coumarin, Sulfadimidine, Moracin D and Salfredin displayed more fungicidal activity than anti-biofilm, and were therefore eliminated in the primary screening. Only two compounds namely 11-amino undeconoic acid and 3HC were found to inhibit the *C. albicans* biofilm without substantially affecting the growth. Among these two potent biofilm inhibitors, 3HC was found to be more significant (p < 0.05) and henceforth, 3HC was chosen to examine its efficacy to inhibit biofilm and to study its influence on virulence factors of *C. albicans*.

### Validation of non-fungicidal anti-biofilm potentials of 3HC against *C. albicans*

**Figure 2:**
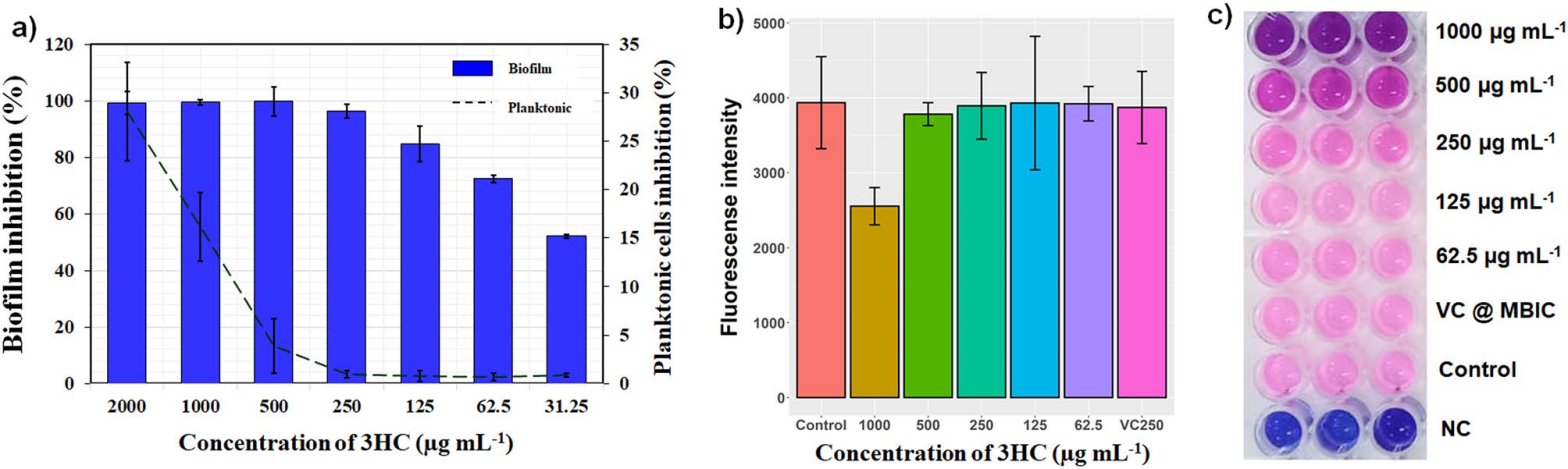
Determination of minimal biofilm inhibitory concentration (MBIC) and non-fungicidal concentration of 3-hydroxy coumarin (3HC). MBIC denotes maximum biofilm inhibition with negligible loss in planktonic cells. (**a**) A dose-dependent reduction biofilm was observed upon treatment with 3HC. The concentration of 250 µg mL^−1^ was chosen as MBIC as biofilm formation was hindered > 95% without any loss in the planktonic growth. (**b**) Measurement of *C. albicans* metabolic viability after 24 h growth in the presence and absence of 3HC, using Alamar blue. The image shows a significant reduction in cell viability at 1000 µg mL^−1^ (MIC) and no reduction in viability at sub-MICs. Error bars indicate the mean values of three experimental triplicates. (**c**) Representative 96-well microtiter plate stained with Alamar blue displaying the true metabolic state of 3HC-treated *C. albicans* cells.

Initially, the non-fungicidal activity of 3HC was confirmed by determining MIC. Among the four tested fungal strains, the planktonic cells of *C. albicans* ATCC reference strain were the most susceptible to the 3HC, followed by the other three clinical isolates. However, the susceptibility was observed to be insignificant, and hence, the MIC of all the studied strains was considered to be 1000 µg mL^−1^. We observed nearly 50% visible cell death at 1000 µg mL^−1^ and further results revealed that the growth of *C. albicans* was not significantly (p > 0.05) inhibited at lower concentrations (Fig. 2a). This was further substantiated by testing the metabolic viability of the cells in the presence and absence of 3HC using Alamar blue (Fig. 2b).

Metabolic viability is a visual observation assay, where Alamar blue, a resazurin dye (non-fluorescent blue coloured dye) is reduced to resorufin (pink coloured fluorescent dye) by metabolically active cells. Hence, the viable cells turn pink, and the non-viable cells remain blue. The results revealed significant (p < 0.05) reduction in cell viability only at MIC (1000 µg mL^−1^), whereas the fluorescent intensity remained unchanged at sub-MICs. The fluorescence intensity of the cells treated with 500 µg mL^−1^ remained nearly the same as the control, and at a concentration of 250 µg mL^−1^, the cells remained completely viable (Fig. 2b,c). All the obtained results confirmed that the 3HC does not possess any fatal impact on the growth and metabolic viability of *C. albicans* below sub-MICs. Hence, the concentrations below sub-MICs (62.5 to 500 µg mL^−1^) were taken further to evaluate antibiofilm potential.

**Figure 3:**
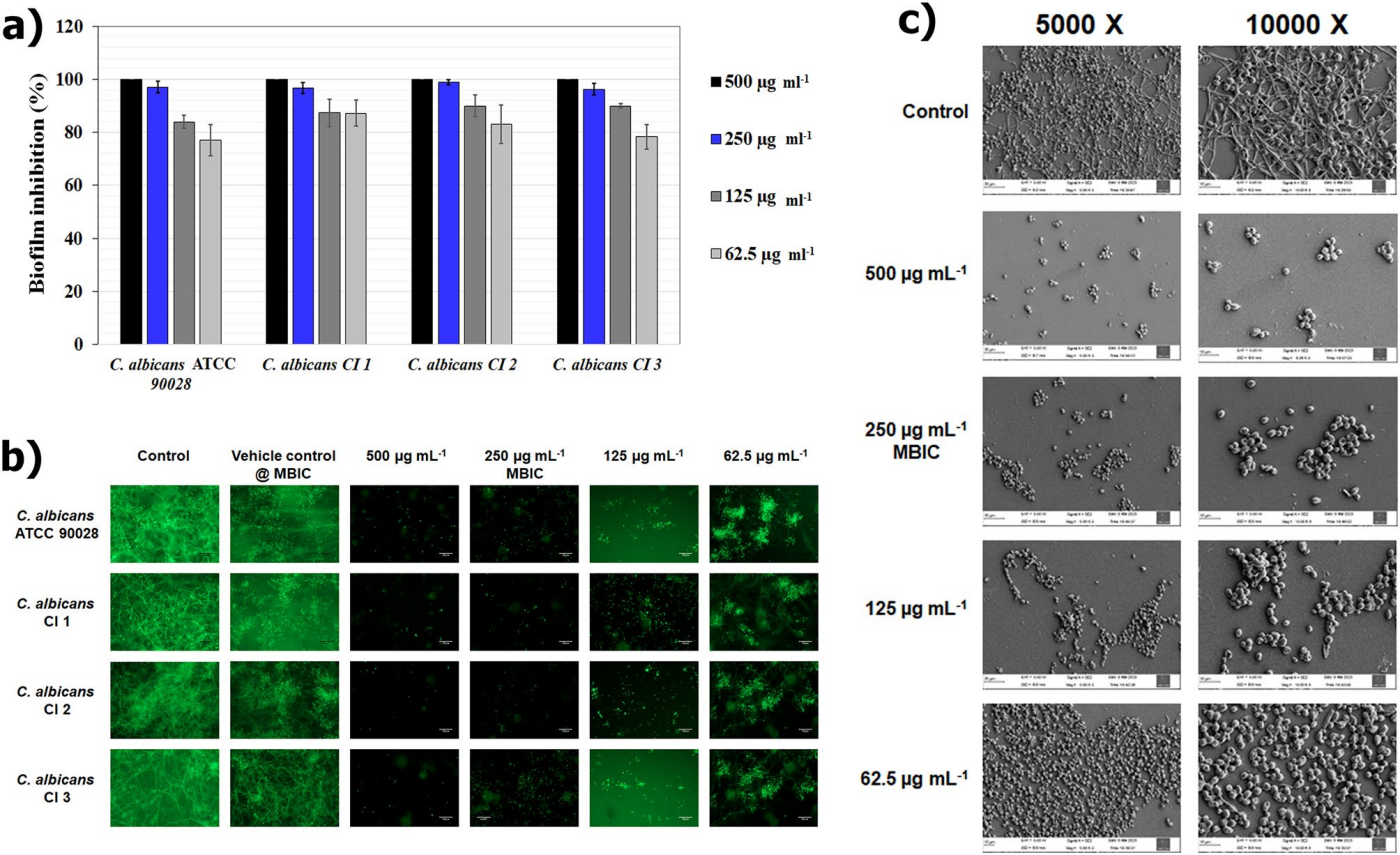
Anti-biofilm efficacy of varying 3-hydroxy coumarine (3HC) concentrations against reference and clinical isolates of *C. albicans*. (**a**) Effect of 3HC on biofilm inhibition of *C. albicans* strains. The biofilm formation of all the four tested *C. albicans* isolates were hindered > 90% at MBIC (blue bar) of 3HC. Error bars represent standard deviation from the mean (n = 9). (**b**) Effect of different concentrations of 3HC on metabolic viability of biofilms formed by various *C. albicans* strains (×200 magnification). A dose dependent reduction in biofilm viability and was observed in all the four isolates of *C. albicans*. Treatment at MBIC had significant reduction in biofilm biomass viability. (**c**) Representative FESEM images of untreated *C. albicans *90028 and those exposed to 3HC at 2 MBIC, MBIC, ½ MBIC and ¼ MBIC showing complete dose-dependent biofilm reduction and complete inhibition of hyphal formation.

After confirming the non-fungicidal activity of 3HC, anti-biofilm activity was examined. The four *C. albicans* strains were tested for their ability to produce biofilms. After 48 h of incubation in the Spider medium, except for *C. albicans* C1, all the other three studied strains had the strong biofilm-forming potential. Similarly, the mixed combination exhibited a robust ability to form biofilms. The CV staining revealed the potential of 3HC to mitigate the biofilm-forming ability of *C. albicans* (Fig. 3a and Supplementary Fig. S3). The 3HC at 125 µg mL^−1^, 250 µg mL^−1^, and 500 µg mL^−1^, concentrations inhibited a maximum of 72%, 98%, and 100% of *C. albicans* biofilms, respectively. However, 3HC at 500 µg mL^−1^ concentration displayed a slight (8%) anti-fungal activity, whereas at 250 µg mL^−1^, it had no effect on the growth of all tested *C. albicans* strains, and therefore growth curves were found similar to those of corresponding controls (Supplementary Fig. S4). Therefore, after carefully evaluating the overall obtained results, 250 µg mL^−1^ was fixed as MBIC and was taken forward to investigate its efficacy on the virulence attributes of *C. albicans*. In the case of artificial saliva, it was observed that even the minimal concentration of 3HC exhibited complete inhibition of biofilm formation of *C. albicans* strain 90028 (Supplementary Fig. S5).

Further, FDA method was used to detect the metabolic activity of the biofilms. As shown in Fig. 3b, the MBIC significantly reduced the cell activity of 48 h biofilms of all the *C. albicans* strains. Control and vehicle control showed dense populations with elongated hyphal cells, whereas the treated groups had hardly any cells. Moreover, we observed scantly no hyphal protrusion in any of the observed cells. The 62.5 µg mL^−1^ treated cells were found to have stunted growth of hyphal elongations (Fig. 3b). These observations were further confirmed using FESEM analysis (Fig. 3c), which confirmed the complete inhibition of hyphal elongations and a concentration-dependent reduction in biofilm formation of *C. albicans* 90028.

### Virulence attributes of *C. albicans* were mitigated upon 3HC treatment

**Figure 4:**
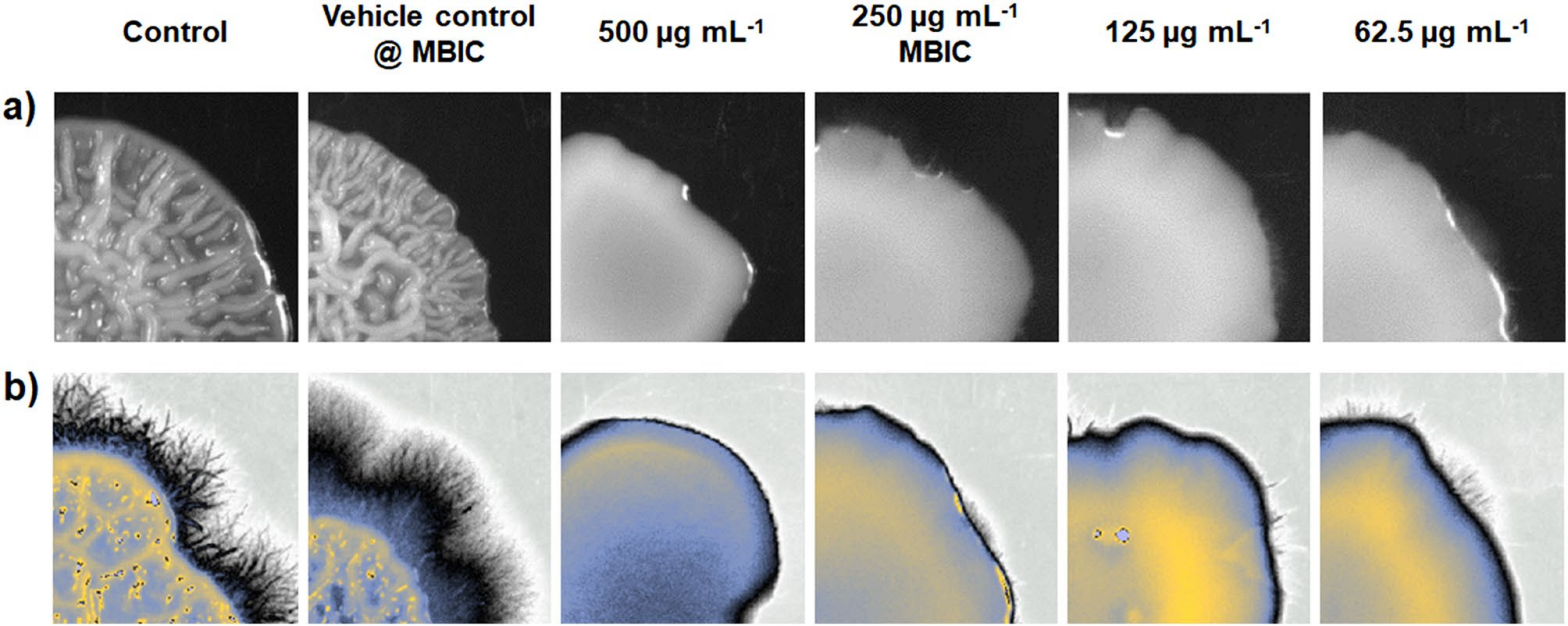
Inhibition of primary virulence factors (filament growth and wrinkle morphology) by the treatment of 3HC. Representative images of Spider medium agar plates show colony morphology exposed to 2 MBIC, MBIC, ½ MBIC and ¼ MBIC of 3HC. After the treatment the colony appeared to have (**a**) smooth surface and (**b**) reduced filament growth compared to that of their respective control. False colour has been applied to the images (**b**) to represent the results clear.

Virulence traits such as filamentous morphology, wrinkle morphology, yeast-to-hyphal transition, and hyphal-to-yeast transition were studied on both control and treated cells. When grown on Spider media supplemented with FBS, the colony of *C. albicans* tend to have a rough surface with wrinkled morphology, and after 3–4 days of incubation, they form filaments (hyphal growth) (Fig. 4a, b). However, the 3HC-treated colonies at MBIC were found to have smooth-edged colonies and were devoid of wrinkle (Fig. 4a and Supplementary Fig. S6) and filamentous growth (Fig. 4b). A reduction in both wrinkle and filamentous growth was also observed at lower 3HC doses (125 µg mL^−1^ and 62.5 µg mL^−1^). Though the appearance of filaments were observed at lower concentrations, their incubation for a longer duration did not promote any further growth and/or elongation of filaments.

### Microscopic analysis revealed the deterioration of surface adherence and hyphal development by *C. albicans* upon treatment with 3HC

**Figure 5:**
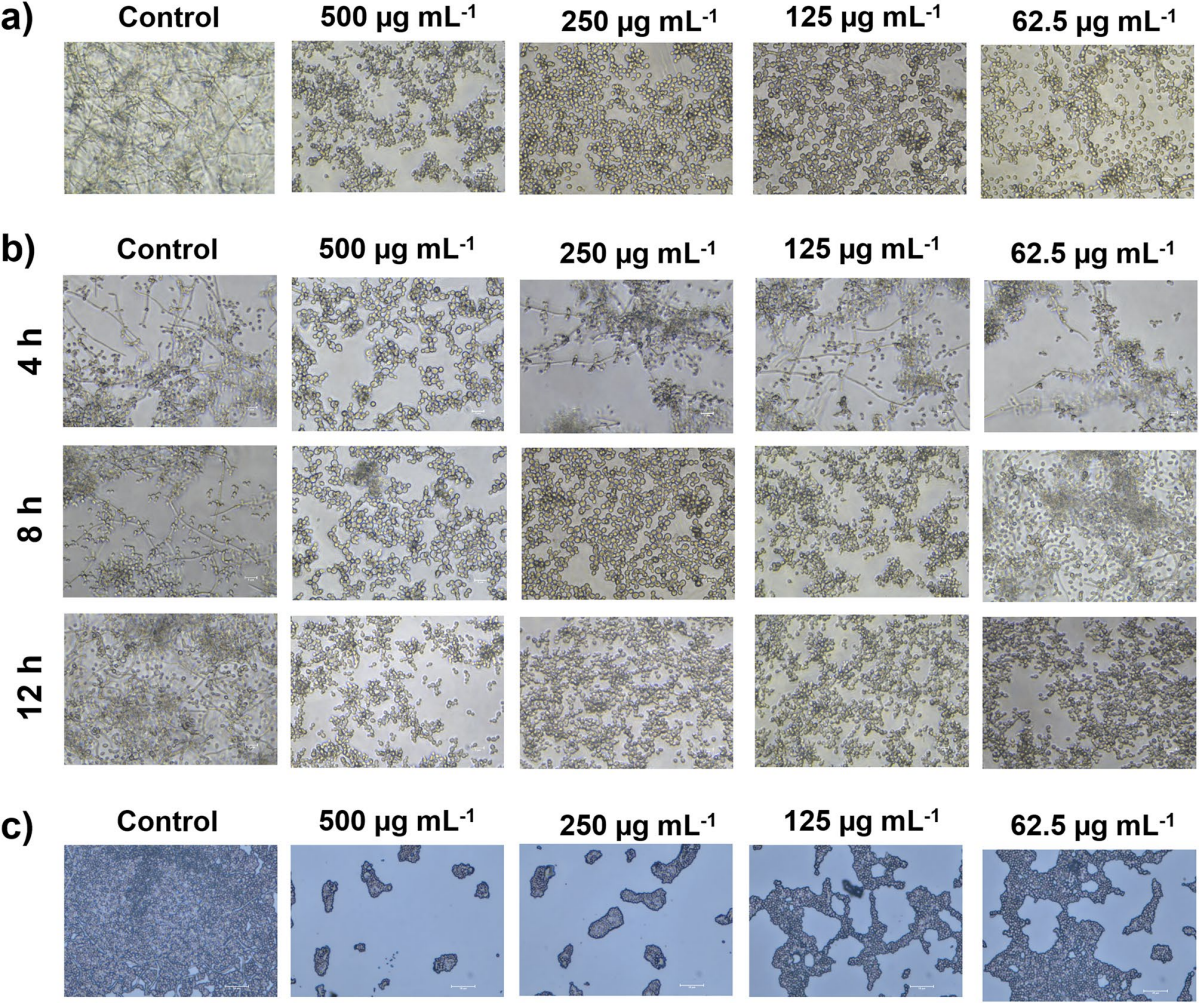
Efficacy of 3-Hydroxy coumarin (3HC) on the morphological transition and mature biofilm disintegration of *C. albicans* cells. Microscopic images showing (**a**) inhibition of hyphal formation and (**b**) reversion of preformed hyphae to yeast cells at 2 MBIC, MBIC, ½ MBIC and ¼ MBIC. (**c**) Disintegration of mature biofilm by 3HC at various concentrations. Maximum dislodging of the pre-formed biofilm was observed at 2 MBIC. Each experiment was repeated at least three times and representative images are presented here.

The prerequisite for the *C. albicans* biofilm formation, hyphal transition, also responded similarly. A complex and highly entangled hyphal network was observed in the case of control cells, whereas 3HC-treated cells (at MBIC) exhibited dispersed yeast cells with no hyphal protrusions. Light microscopy images of control and 3HC-treated cells clearly showcased a reduction in *C. albicans* surface adherence and hyphal development (Fig. 5a). The thickness and intensity of the biofilm and the ability of the cells to shift from yeast-to-hyphal mode were significantly reduced. The reduction in hyphal transition was found even with the minimum concentration (62.5 µg mL^−1^) of 3HC. Control cells were found to be structurally stable with complex hyphal protrusions.

In addition, the ability to reverse hyphal-to-yeast transition is also a much-needed property of a potent anti-biofilm compound. Hence, the capability of 3HC to reverse the preformed hyphal cells was tested by growing cells in the RPMI medium, which facilitates hyphal growth. Continuous monitoring of 3HC treated cells at 4 h intervals revealed that the reversion of hyphal-to-yeast was initiated after 4 h of 3HC treatment (Fig. 5b). Moreover, 3HC also exhibited both dose-dependent and time-dependent reversibility. For example, treatment with MBIC caused a reversal of hyphal-to-yeast transition after 8 h (~ 90%), while at 62.5 µg mL^−1^, the maximum reversion was observed after 12 h.

### 3HC disintegrates mature *C. albicans* biofilms

Efficiency to disrupt preformed biofilm is a crucial attribute of a potent anti-biofilm candidate. To test this ability, *C. albicans* 90028 was allowed to form biofilm for 48 h. We observed that 3HC at both MBIC and 2× MBIC had the capability to dislodge surface-attached cells after 24 h of incubation (Fig. 5C).

### Differential expression of virulence genes in *C. albicans* upon 3HC treatment

Since all the assays on physiology demonstrated significant reductions in adhesion and phenotypic switching, those genes which are involved in biofilm formation, hyphal morphogenesis, and yeast-to-hyphal transition were primarily targeted for ascertaining their differential expression through qPCR analysis to validate the ability of 3HC at genome level (Supplementary Fig. S7).

As expected, the results revealed that adhesion-responsible genes such as *als1,* and *eap1* were downregulated significantly (p < 0.05) with a fold change of 6 and 4, respectively. The expression of genes responsible for hyphal and filament development, such as *hst7* and *ume6*, were the most significantly (p < 0.005) downregulated (> 10-fold). Additionally, the downregulation of other adhesion and filamentation-related genes, including *efg1, cph1, eap1, ras1, als1* and *ece1*, further corroborates the in vitro efficacy of 3HC in anti-biofilm, anti-hyphal, and phenotypic switch control (Supplementary Fig. S7).

### Toxicity analyses of 3HC against HBECs and HECs

**Figure 6:**
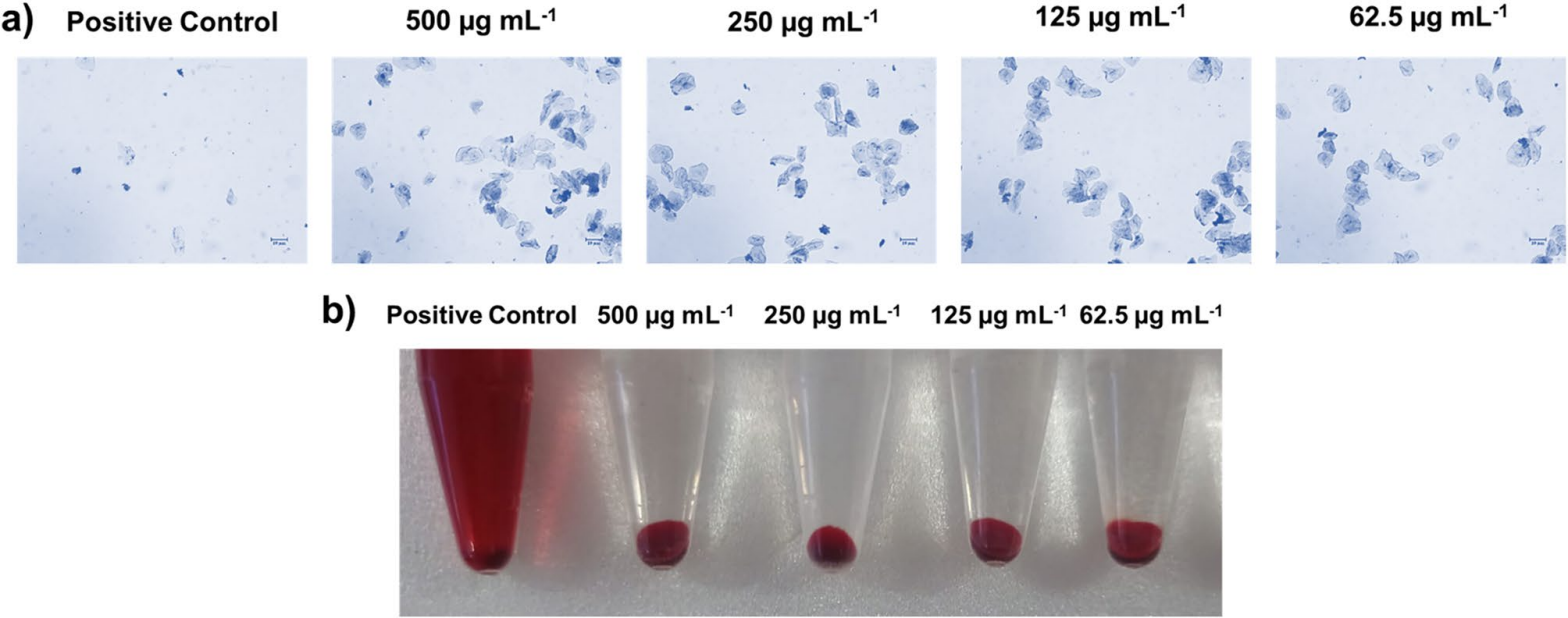
Non-toxic effect of 3HC on (**a**) human buccal epithelial cells (HBECs) and (**b**) human erythrocytes. Hydrogen peroxide and Triton X-100 were used as positive controls for HBECs and erythrocytes, respectively. Both the cells treated with different concentrations of 3HC (62.5 to 500 µg mL^−1^) exhibited no toxic effect, except for the positive control. VC denotes vehicle control (ethanol).

The non-toxic nature of 3HC was evaluated against buccal cavity epithelial cells (Fig. 6a) and erythrocytes (Fig. 6b). In both cells, their respective positive control exerted an adverse effect on the cells. For instance, Triton X-100 caused complete lysis of the erythrocytes, resulting in a red-coloured solution. Similarly, hydrogen peroxide completely disintegrated the structure of HBECs. However, treatment with 3HC did not cause any negative influence on both cases, indicating its non-toxic nature, with no loss in cell structure or integrity.

## Discussion

Globally, *C. albicans* is the leading cause of invasive candidiasis. Several epidemiological studies have shown that the formation of biofilm increases the mortality of invasive candidiasis^41,42^. *C. albicans* is more likely to form biofilms than other *Candida* species such as *C. tropicalis* and *C. glabrata*^43^. Generally, a biofilm formed by *C. albicans* consists of a network of three-dimensional hyphae and an extracellular matrix that is highly dynamic and extremely structured. Compared to their planktonic counterparts, *C. albicans* cells in biofilm are less responsive to commonly used antifungal drugs such as azoles and conventional amphotericin B^44,45^. Due to the infectiveness of commonly used anti-fungal drugs against *C. albicans* biofilm lifestyle, their overuse is widely practiced, which in turn favours the development of multidrug resistance^46^. In addition, multidrug resistance also enhances the recurrence of treatment failures due to the exertion of selective pressure exerted by conventional antifungal drugs^47,48^. Considering the severity of biofilm-related diseases, it is imperative to find alternative antifungal drugs that can efficiently control biofilms without affecting the growth of planktonic cells.

To cope with this, metabolites produced by natural biofilm-forming bacterial consortia were used in this study. Compounds derived from marine bacterial communities are highly valuable in promoting bacterial survival, particularly in competitive environments such as marine biofilms. Screening various bacterial extracts for anti-biofilm compounds revealed the efficacy of most extracts. Interestingly, the biofilm enhancing ability was also observed by few bacterial isolates. This ability signifies the nature of the bacteria and its environment, i.e., the natural biofilm. Generally, bacterial community residing in marine biofilms exhibit both synergistic and competitive behaviour^49^. Synergistic behaviour promotes the growth of peripheral living communities, whereas competitive behaviour has the opposite effect. Bacterial isolates that showed the biofilm enhancing ability might show cooperative behaviour with other bacterial members. However, to reduce the virulence of potent pathogens, competitive bacterial isolates play a significant role. Compounds produced by these competitive bacterial isolates are widely explored for their anti-biofilm, antimicrobial, and anti-quorum sensing properties against both Gram-positive and -negative bacteria^50–53^. However, the efficacy of marine bacterial-derived metabolites (particularly from marine biofilms) against the human fungal pathogen, specifically *C. albicans* is rarely studied. In this backdrop, the present study investigated the in vitro inhibitory efficacy of 3HC produced by a marine bacterium *B. abyssalis* against the biofilm and virulence factors of *C. albicans*.

Coumarin and its derivatives are natural polyphenolic crystalline oxygenated heterocyclic compounds that are predominantly found in plants as heterosides or in free form^54^. Although they are mostly plant-associated, some derivatives, such as novobiocin, coumermycin, and aflatoxin have also been found to be produced by microbes^55,56^. The compound of interest in our study (3HC) is a simple coumarin that has received little attention. Recently, 3HC was identified for its ability to inhibit recombinant human tyrosinase and was effectively engineered for topical pharmaceutical applications^57^. Nevertheless, 3HC has not been studied against any human pathogen, and therefore, this study is the first to identify 3HC from a marine bacterium and to determine its anti-biofilm efficacy against *C. albicans*.

The entire biofilm development of *C. albicans* comprises three main phases, namely the early, developmental, and mature stages^58^. In the present study, a significant inhibitory effect of 3HC against *C. albicans* biofilm was observed. Importantly, 3HC suppressed about 72% of the biofilm formation at concentration of 125 µg mL^−1^ and completely inhibited (~ 98%) the biofilm formation at 250 µg mL^−1^ concentration. Changes in the intensity of CV staining in both spectroscopic and light microscopic studies confirmed this observation. Further, the anti-biofilm effect of 3HC was noted to be dose-dependent. Unlike planktonic cells, biofilm-forming cells exhibit unique characteristics and are encased within a protective extracellular matrix. The MBIC represents the concentration of 3HC needed to inhibit the formation and growth of biofilms, rather than eradicating the entire microbial population within the biofilm. It serves as an indicator of the compound's efficacy in managing biofilm-related complications by impeding biofilm development. This approach acknowledges the unique characteristics of biofilms and aims to mitigate their detrimental effects, such as reduced susceptibility to antibiotics and enhanced pathogenicity, rather than focusing solely on microbial killing. The high hydrophobic nature of 3HC would have prevented the cells to adhere on the surface^57^. In addition, an ideal anti-biofilm compound should also be non-fungicidal to prevent the chances of resistance development^59^. Based on the viability assay results, 3HC was shown to have non-fatal effects, further strengthening its effectiveness as a potent anti-biofilm agent. In addition, the complete inhibition of biofilm formation in the case of artificial saliva could be attributed to its acidic nature. Artificial saliva formulations typically strive to replicate the slightly acidic pH (ranging from 6.2 to 7.6) of natural saliva. This acidity plays a crucial role in establishing an environment that is less favourable for the growth of *C. albicans*. Moreover, the introduction of 3HC might have further enhanced the inhibition of biofilm formation, resulting in a more effective inhibition.

Adherence of *Candida* cells to any surface is the primary step in biofilm formation cascade. After adhesion onto a surface, the yeast cells gradually develop into hyphal forms, which is considered highly important to both biofilm formation and dissemination^60^. Moreover, cell adhesion and yeast-to-hyphal transition are the crucial pathogenic elements in *C. albicans*, which are known to actively elicit the invasion of the *Candida* cells into the host^60^. This was discovered in a study by Saville et al.^61^, where a group of mice was injected with a modified *C. albicans* strain that could be externally modulated for yeast-to-hyphal morphology transition. They achieved this by placing the negative regulator of the filamentation gene; *nrg1* under the control of a tetracycline regulatable promoter and found that mice injected with this strain succumbed to infection, while the control did not^62^. Hence, inhibiting these pathogenic elements induces a defect in biofilm formation, which is a key target in biofilm-specific treatments^63,64^.

The filamentation assay showed that 3HC obviously inhibited the morphological transition of *Candida albicans* cells. Starting at 62.5 µg mL^−1^ of 3HC, a large number of cells were locked in the yeast form and at higher concentrations, 3HC completely inhibited the filamentous growth. Similarly, after cell adhesion and growth, the colony's surface wrinkles increase its surface area to acquire both nutrients and for further hyphal development and adhesin production^65^. The active compound, 3HC even at its lowest concentration (62.5 µg mL^−1^) restricted the colony surface wrinkle formation. Prevention of these virulent factors that are highly necessary for the invasion, attachment, and biofilm formation validates that 3HC could evidently impede the infections of *C. albicans*.

Besides acting against the virulence factors for early biofilm initiation, 3HC was also found to act effectively on mature biofilm and virulence factors. For instance, 3HC potentially induced the transition from the hyphae-to-yeast-cells and significantly disrupted mature biofilm. Such a transition is advantageous in cases of mature infection, where inhibiting the virulence factors can increase the susceptibility of the *Candida* cells to the host immune response and therapeutics.

Further, to elucidate the plausible anti-biofilm mechanism of 3HC, qPCR was performed and changes at transcription levels were determined. The genes were selected based on the positive regulation of initial virulence factors such as adhesion and morphogenesis. The results revealed the negative regulation of the genes involved in virulence, including *als3, cph1, eap1, efg1, hst1, hwp1, ras1*, and *ume6*. The *als* family of genes, including *als3*, play a crucial role in the adhesion of yeast cells and is an integral part of biofilm formation. Other transcriptional factors such as *efg1* and *cph1* are the first identified regulators of hyphal development and synergistically regulate virulence genes^66^. Hyphal-specific transcription factor *hwp1* regulates hyphal development, mating efficiency and biofilm integrity. The downregulation of *hwp1* and *als3* observed in this study indicate the reduction of the cell adhesion and virulence ability, which could further cause the detachment of biofilms from abiotic substrates^66,67^. The downregulation of genes such as *ume6* and *ras1*, which are responsible for morphological switching (yeast-to-hyphal) and polarized filaments, respectively, upon 3HC treatment confirms its anti-biofilm potential^68^. Overall, our qPCR results concluded that 3HC impedes the most crucial primary virulence factors, including adhesion and morphogenesis that are required for biofilm formation by *C. albicans* cells.

Despite all the above discussion, for clinical applications, a compound must be non-toxic to human cells. Here, human erythrocytes and HBECs are targeted concerning both intestinal and oral candidiasis. The results showed no toxicity effect by the active compound 3HC, confirming its potency for clinical applications. Moreover, a previous study has also reported the non-cytotoxic effect of 3HC on B16F10 melanoma cells^57^.

In conclusion, this study demonstrated, for the first time, the anti-biofilm and anti-virulence efficacy of 3HC, a marine bacterium-derived compound, on *C. albicans*. This compound prominently impaired the initial adherence of *C. albicans* and inhibited the morphological transition of yeast cells. Compound 3HC not only specifically inhibited biofilm formation and hyphal development but also impedes the expression of genes that are positively involved in initial adhesion, morphogenesis, and biofilm formation in *C. albicans*. The results of toxicity experiments on human erythrocytes and HBECs confirmed the innocuous nature of 3HC. Given its prominent anti-biofilm and anti-virulence activity against *C. albicans* with non-fatal and non-toxic nature, 3HC can be considered a promising compound to evade the onset of biofilm-mediated infections caused by *C. albicans*. Overall, the present study defines new insights for the identification of novel bioactives from a highly competitive environment such as marine biofilm. In future studies, deciphering detailed molecular mechanisms of 3HC using transcriptomics and proteomics approaches would be a significant scientific contribution.

## Supplementary Information

Supplementary Information.

## Data Availability

The data supporting the finding of this study are provided within the manuscript. The bacterial 16S rRNA gene sequencing read generated during the current study is available in NCBI’s GenBank under accession number MZ357100.
