# Supplementary material for: 3-Hydroxy coumarin demonstrates anti-biofilm and anti-hyphal efficacy against Candida albicans via inhibition of cell-adhesion, morphogenesis, and virulent genes regulation
Source: Sci Rep. 2023 Jul 19;13:11687. doi: 10.1038/s41598-023-37851-1 (PMC10356798; doi:10.1038/s41598-023-37851-1)

Supplemental Material for the manuscript entitled "3-hydroxy coumarin demonstrates anti-biofilm and anti-hyphal efficacy against *Candida albicans* via inhibition of cell-adhesion, morphogenesis, and virulent genes regulation"

T J Sushmitha[a, †], Meora Rajeev[a, †,#], Vellaisamy Kathirkaman[a], Singh Shivam[a], Toleti Subba Rao[b], Shunmugiah Karutha Pandian[a,*]

[a] Department of Biotechnology, Alagappa University, Science Campus, Karaikudi 630 003, Tamil Nadu,

[b] School of Arts & Sciences, Sai University, OMR, Paiyanur-603105, Tamil Nadu, India.

#Current affiliation: Department of Biological Sciences and Bioengineering, Inha University, Inharo 100, Incheon 22212, Republic of Korea.

*Corresponding Author's E-mail address: sk_pandian@alagappauniversity.ac.in

[†] These authors contributed equally to this work.

Supplementary Table

Table S1. List of compounds identified by HR-LCMS analysis in cell-free culture supernatant (CFCS) of a marine bacterium *Brevundimonas abysallis* isolated from early-stage marine biofilms.

S. No.	Identified compound name	Retention time (min)	Mass
1	11-amino-undecanoic acid	1.273	201.1719
2	3-Hydroxycoumarin	11.232	162.0313
3	Armillarin	15.138	414.2042
4	6-Deoxyjacareubin	14.533	310.0838
5	Rabelomycin	12.909	338.0781
6	4-(1,2-Epoxyethyl)- 8,9-epoxy-enediyne	11.72	208.052
7	4-Hydroxy-8- methoxy-2H-furo[2,3-h]-1-	8.546	232.0367
8	(+)-Veraguensin	13.166	372.1929
9	Tetracenomycin M	16.054	356.0918
10	Glycyrrhizaisoflavone A	13.351	370.1067
11	Sulfadimidine	13.577	278.0859
12	Axisothiocyanate 3	5.097	263.1754
13	Moracin A	12.807	286.0858
14	Amobarbital	5.815	226.1313
15	Dihydro-O-	16.432	340.0968
16	Triphenyl phosphate	15.985	326.0705
17	Beclomethasone	13.169	408.1696

18	para- Trifluoromethylphenol	17.293	162.0298
19	Epithienamycin E	1.357	392.0376
20	1-(2,6,6-Trimethyl-2-cyclohexen-1-yl)-1,6-heptadien-3-one	13.228	232.1782
21	(+/-)-3-[(2-methyl-3- furyl)thio]-2-butanone	0.815	184.0577
22	Sulfoxone	12.034	404.0141
23	Butoctamide	4.844	315.204
24	(6beta,8betaOH)-6,8-Dihydroxy-7(11)-eremophilen	17.41	266.1523
25	Alizapride	6.288	315.1679
26	Citric acid	1.531	192.0258
27	Solanocapsine	8.218	430.3535
28	4,4’-Diapophytoene	8.904	408.3727
29	4-Methylumbelliferyl	9.786	218.0571
30	Isoferulic acid	9.869	194.0572
31	Pinacidil	10.43	245.1623
32	Salfredin B11	11.166	232.0729
33	2’,4’-Dihydroxy-2-biphenylcarboxylic acid	11.174	230.0571
34	Salfredin B11	11.479	232.0729
35	2,3-Dihydro-3-hydroxy-6-methoxy-2,2-dimethyl-4H-1-benzopyran-4	12.7	222.0888

36	Momilactone A	13.226	314.1898
37	Moracin A	14.042	286.086
38	3-(1-Pyrrolidinyl)-2-	1.379155.130	155.1304
39	Fluticasone	15.622	500.1838
40	Tetracenomycin D1	17.239	336.0633
41	Isocitrate	1.525	192.0261
42	Cotinine	1.576	176.0945
43	6,8-Dihydroxypurine	1.638	152.0327

Supplementary Figures

Figure S1. Screening of the cell-free culture supernatant (CFCS) of bacterial isolates obtained from early-stage marine biofilm from a) day 1, b) day 9, c) day 6, d) day 12 and e) day 15 for anti-biofilm potential against *C. albicans*. Error bars represent standard deviations from the mean (n = 3).

Figure S1a)

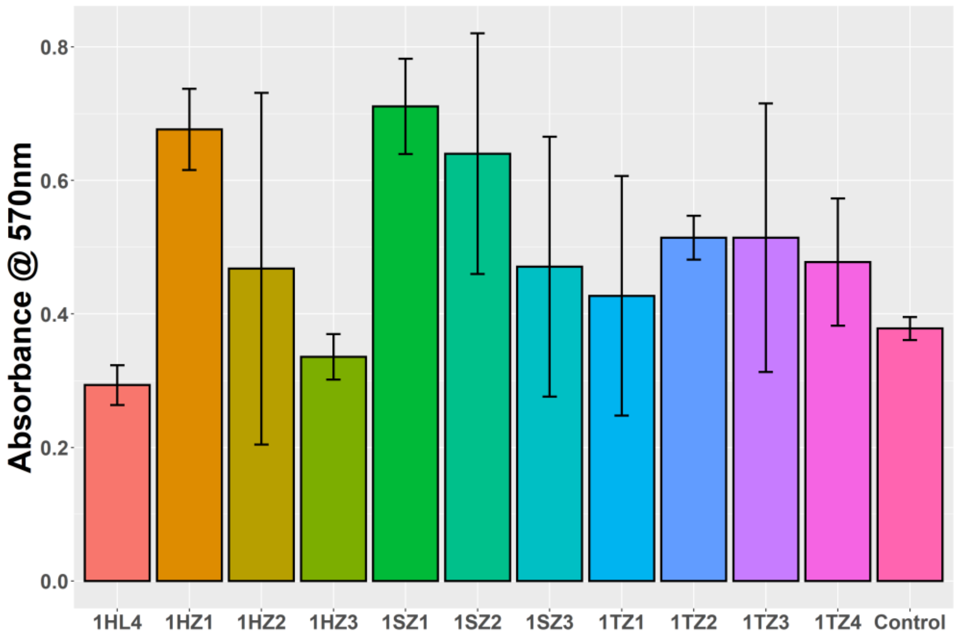

Figure S1b)

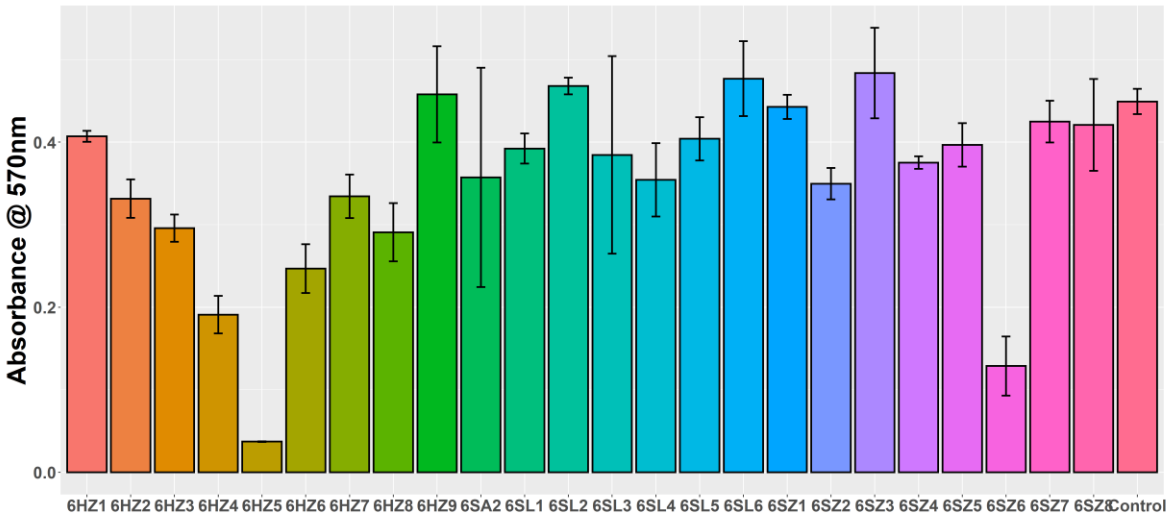

Figure S1c)

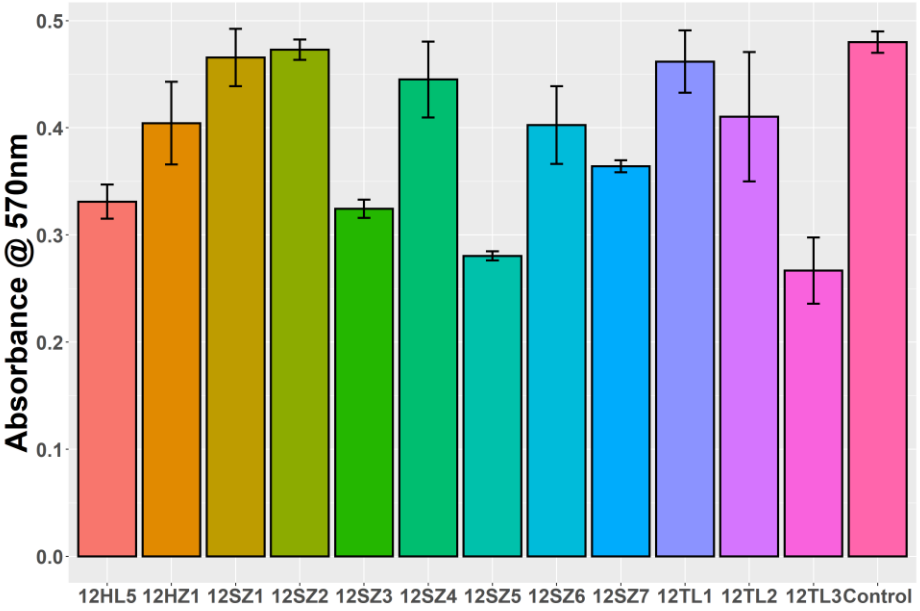

Figure S1d)

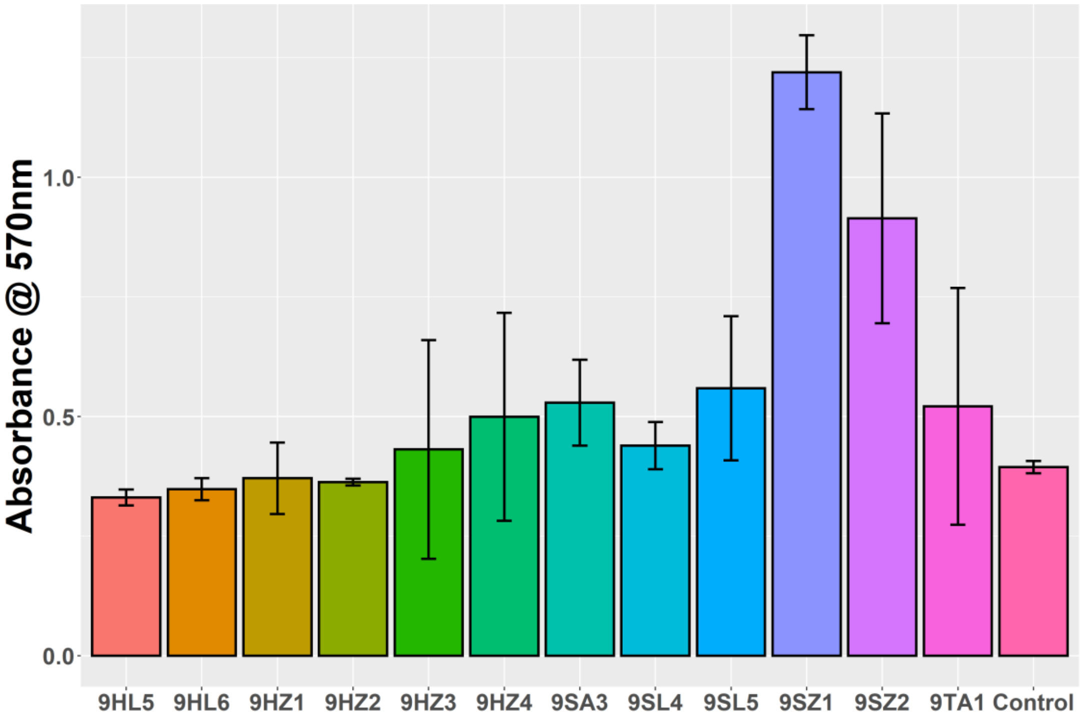

Figure S1e)

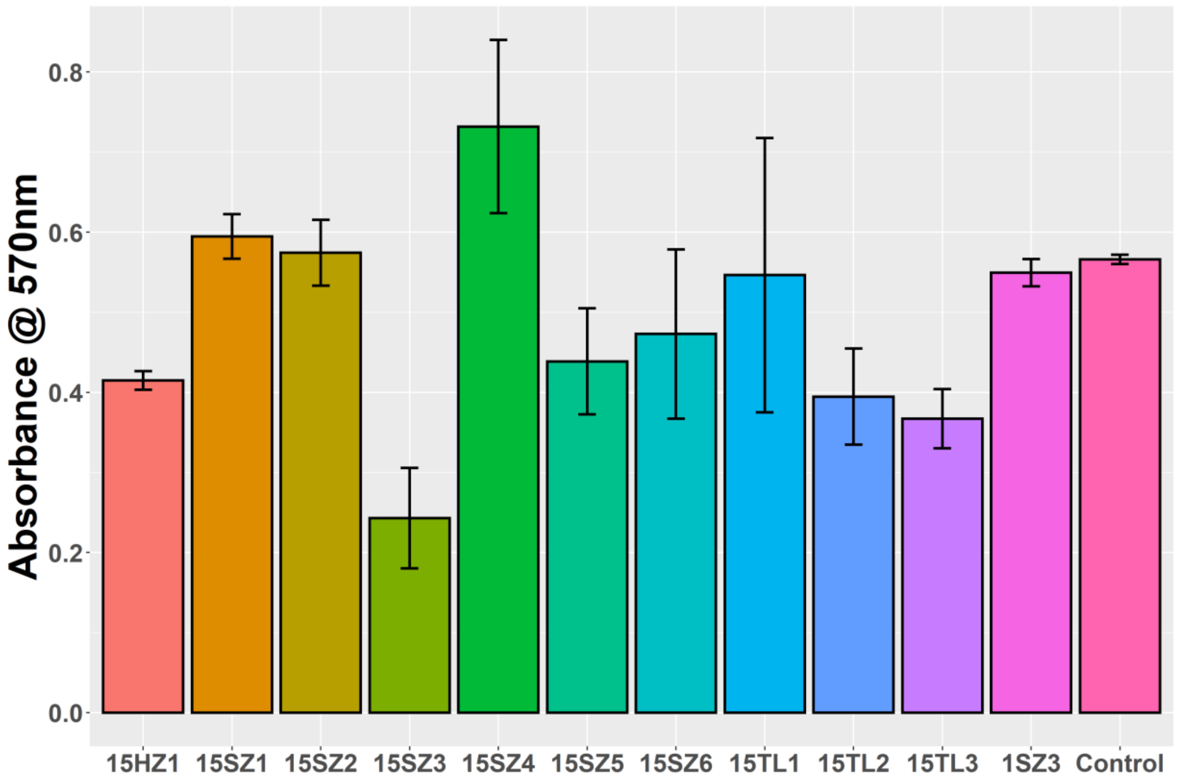
Absorbance @ 570nm
0.0
0.2
0.4
0.6
0.8
15HZ1
15SZ1
15SZ2
15SZ3
15SZ4
15SZ5
15SZ6
15TL1
15TL2
15TL3
1SZ3
Control

Figure S2. Phylogenetic tree showing phylogenetic relation of *Brevundimonas abysallis* strain 6HZ5 with other selected members of genus *Brevundimonas*. The tree was constructed based on partial 16S rRNA gene sequences using the neighbor-joining method with 1000 bootstrap replicates in MEGA 11 software. GenBank accession numbers of respective isolates are provided before their names. *Escherichia Coli* was used as outgroup and *B. abysallis* strain 6HZ5 used in this study is highlighted. Bold numbers denote the bootstrap values and non-bold denotes the scale bar distance of corresponding branch.

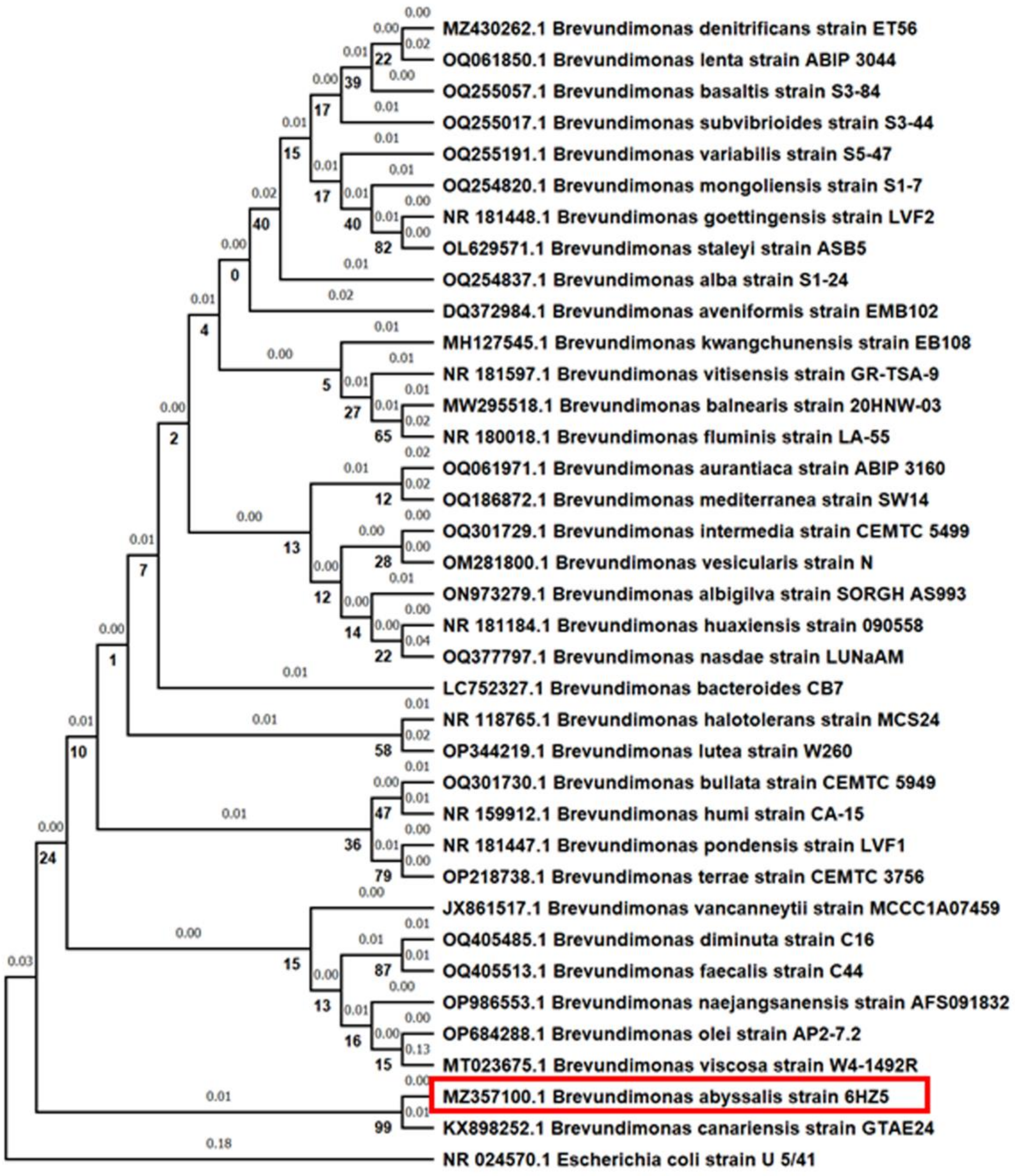

Figure S3. Antibiofilm efficacy of 3HC against reference (*C. albicans* ATCC 90028) and clinical strains (*C. albicans* CI 1, CI 2 and CI 3). Representative result of crystal violet assay in 24-wells MTP showing the dose-dependent reduction in the biofilm of all the investigated *C. albicans* strains.

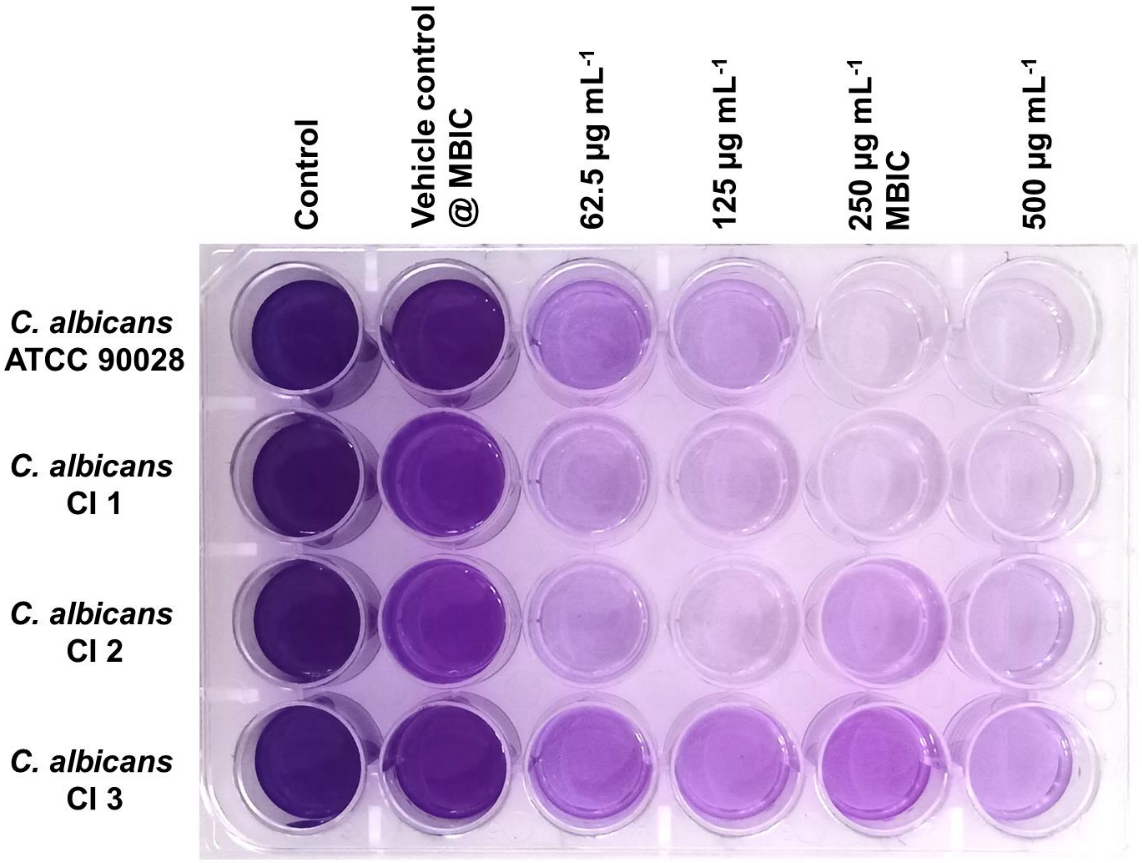

Figure S4. Effect of 3-hydroxy coumarin (3HC) on growth of various *C. albicans* strains. Growth curve of *C. albicans* strains was determined in the absence (positive control; PC) and presence of 3HC at minimal biofilm inhibitory concentration (MBIC; 250 µg/ml).

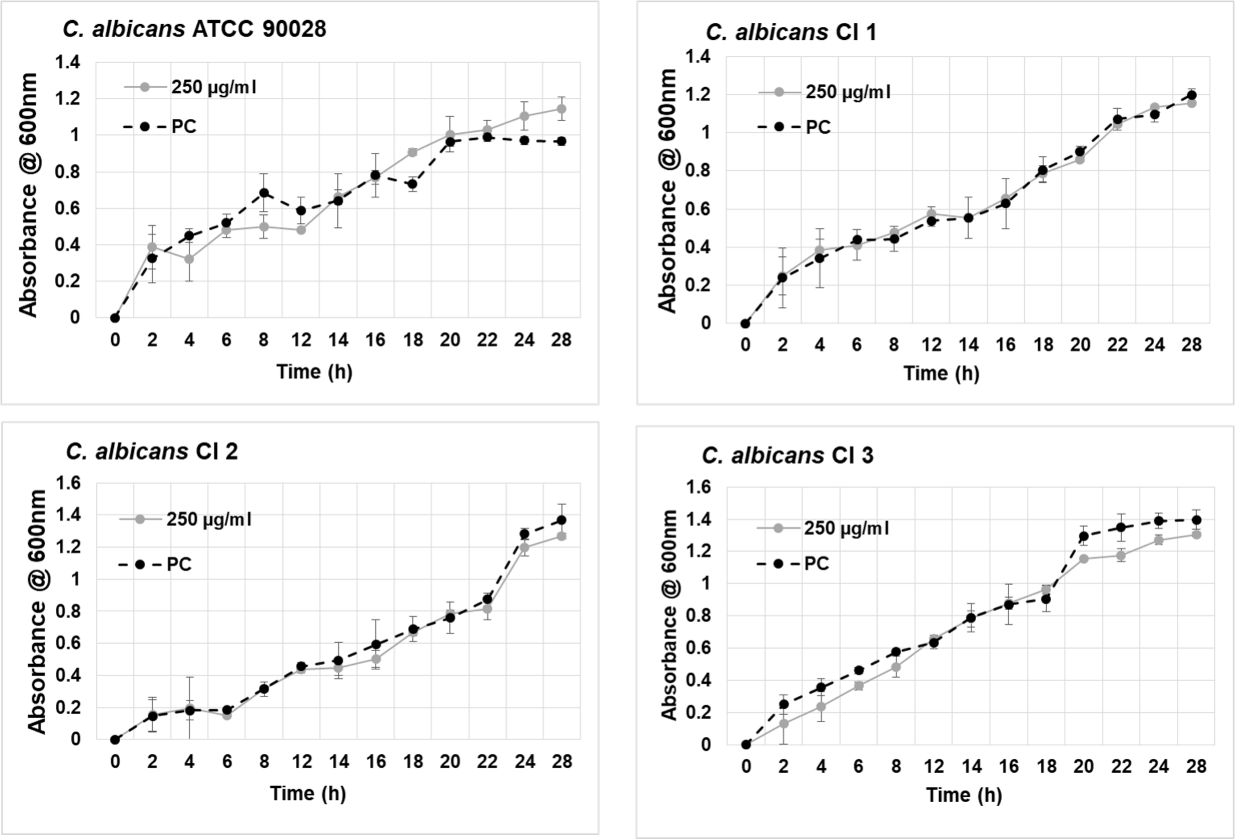

Figure S5. Effect of 3HC in artificial saliva medium. Representative image of 24-well MTP showing the complete inhibition of *C. albicans strain* 90028 biofilms upon various concentration of 3HC treatment.

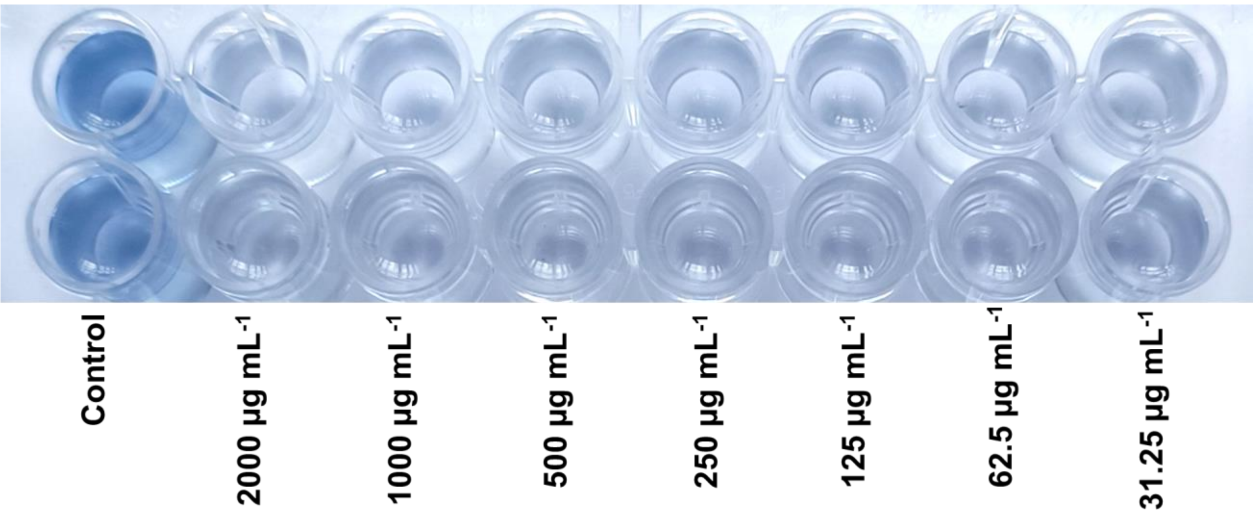

Figure S6. 3HC impeding the primary virulence attributes of *C. albicans*. Representative complete image of *C. albicans* strains showing reduction in wrinkle morphology and filament growth upon treatment with ½ MBIC, MBIC and 2 MBIC.

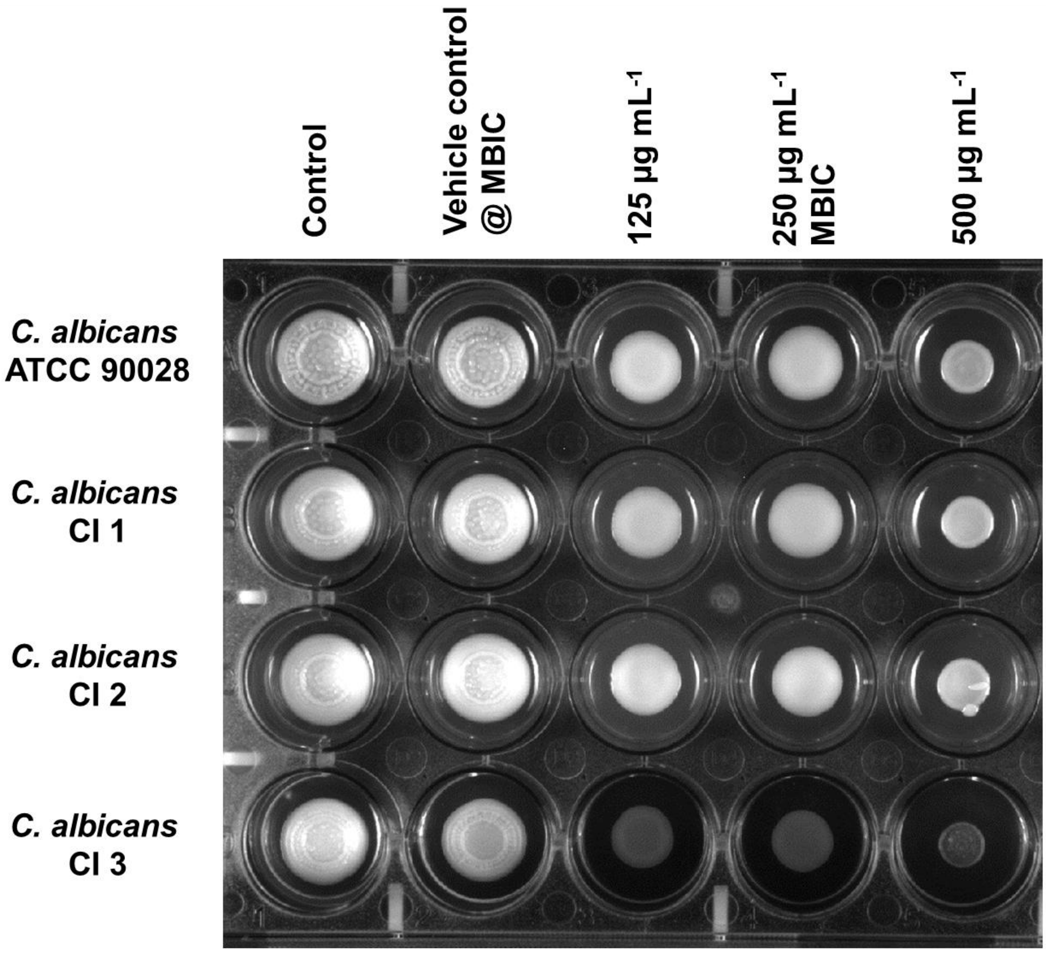

Figure S7. Differential regulation of genes involved in biofilm and hyphal development of *C. albicans* by 3HC treatment at its minimal biofilm inhibitory concentration (MBIC, 250 µg mL^{-1}). Image showing the significant downregulation of genes involved in initial adhesion (*als3*, *eap1*), filamentation (*cph1*, *hst7*, *efg1*, *ume6*), and hyphal growth (*eap1*, *hst7*, *hwp1*, *ras1*) by MBIC of compound 3HC. Error bars represent standard deviations from the mean of three experimental triplicates.

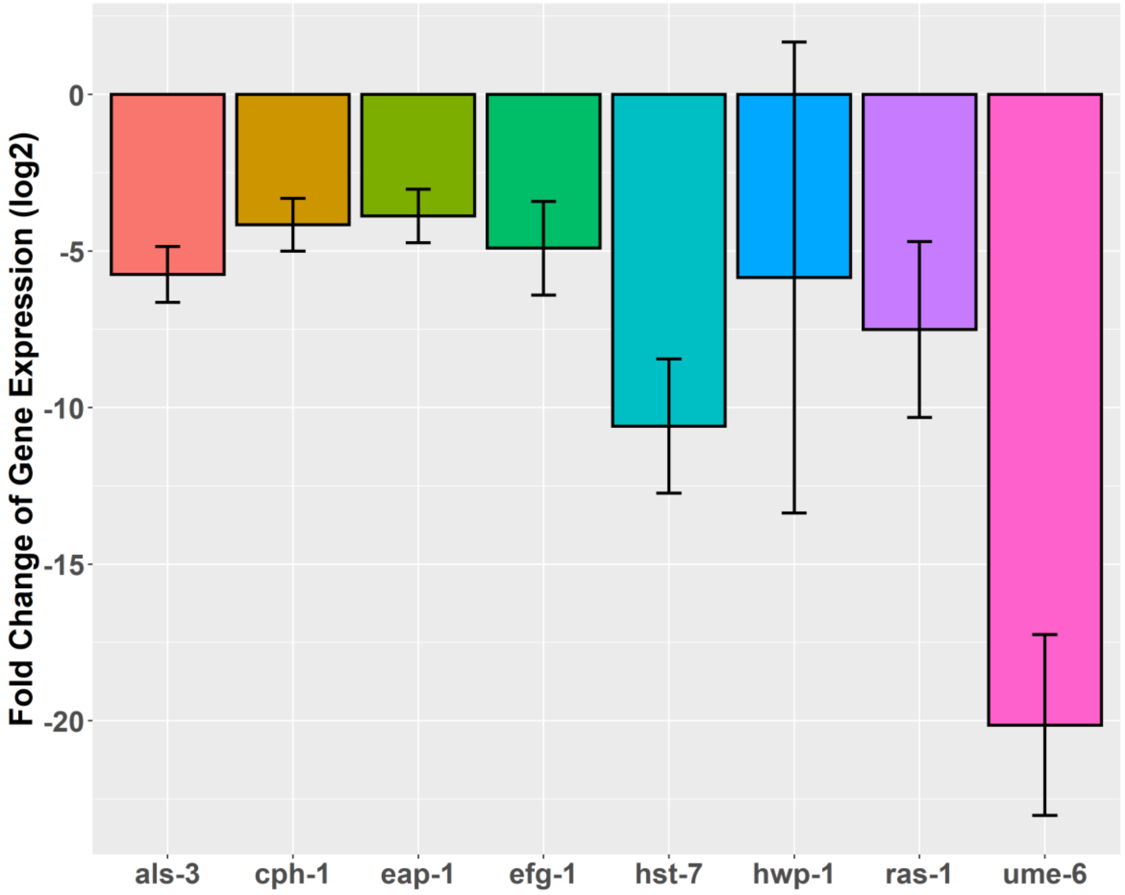

Supplement: Supplementary file 1 — Supplementary Information. [file 41598_2023_37851_MOESM1_ESM.pdf]
